# Dose-dependent alcohol-induced alterations in chromatin structure persist beyond the window of exposure and correlate with fetal alcohol syndrome birth defects

**DOI:** 10.1186/s13072-015-0031-7

**Published:** 2015-09-28

**Authors:** Kylee J. Veazey, Scott E. Parnell, Rajesh C. Miranda, Michael C. Golding

**Affiliations:** Room 338 VMA, 4466 TAMU, Department of Veterinary Physiology, College of Veterinary Medicine and Biomedical Sciences, Texas A&M University, College Station, TX 77843-4466 USA; Bowles Center for Alcohol Studies and Department of Cell Biology and Physiology, School of Medicine, CB# 7178, University of North Carolina, Chapel Hill, NC 27599 USA; Texas A&M Health Sciences Center, Texas A&M University, 8441 State Highway 47, Clinical Building 1, Suite 3100, Bryan, TX 77807 USA

**Keywords:** Developmental programming, Epigenetics, Fetal alcohol syndrome, Environmental epigenetics, Chromatin, Teratogen, Neurodevelopmental programming, Neural stem cells, Epigenetic inheritance

## Abstract

**Background:**

In recent years, we have come to recognize that a multitude of in utero exposures have the capacity to induce the development of congenital and metabolic defects. As most of these encounters manifest their effects beyond the window of exposure, deciphering the mechanisms of teratogenesis is incredibly difficult. For many agents, altered epigenetic programming has become suspect in transmitting the lasting signature of exposure leading to dysgenesis. However, while several chemicals can perturb chromatin structure acutely, for many agents (particularly alcohol) it remains unclear if these modifications represent transient responses to exposure or heritable lesions leading to pathology.

**Results:**

Here, we report that mice encountering an acute exposure to alcohol on gestational Day-7 exhibit significant alterations in chromatin structure (histone 3 lysine 9 dimethylation, lysine 9 acetylation, and lysine 27 trimethylation) at Day-17, and that these changes strongly correlate with the development of craniofacial and central nervous system defects. Using a neural cortical stem cell model, we find that the epigenetic changes arising as a consequence of alcohol exposure are heavily dependent on the gene under investigation, the dose of alcohol encountered, and that the signatures arising acutely differ significantly from those observed after a 4-day recovery period. Importantly, the changes observed post-recovery are consistent with those modeled in vivo, and associate with alterations in transcripts encoding multiple *homeobox* genes directing neurogenesis. Unexpectedly, we do not observe a correlation between alcohol-induced changes in chromatin structure and alterations in transcription. Interestingly, the majority of epigenetic changes observed occur in marks associated with repressive chromatin structure, and we identify correlative disruptions in transcripts encoding *Dnmt1*, Eed, *Ehmt2* (*G9a*), *EzH2*, *Kdm1a*, *Kdm4c*, *Setdb1*, *Sod3*, *Tet1* and *Uhrf1*.

**Conclusions:**

These observations suggest that the immediate and long-term impacts of alcohol exposure on chromatin structure are distinct, and hint at the existence of a possible coordinated epigenetic response to ethanol during development. Collectively, our results indicate that alcohol-induced modifications to chromatin structure persist beyond the window of exposure, and likely contribute to the development of fetal alcohol syndrome-associated congenital abnormalities.

**Electronic supplementary material:**

The online version of this article (doi:10.1186/s13072-015-0031-7) contains supplementary material, which is available to authorized users.

## Background

From studies using a diverse range of model organisms, we now acknowledge that epigenetic modifications to chromatin structure provide a plausible link between environmental exposures and alterations in cellular function leading to pathology [1]. These revelations create novel perspectives in our understanding of fetal development that must be investigated if we are to fully understand the molecular origins of birth defects. However, for many teratogens the link between exposure and altered epigenetic programming remains poorly defined.

Alcohol consumption during pregnancy is widespread in our society despite its proven association with the development of birth defects and severe mental impairment. Work from a number of independent research groups have demonstrated that ethanol (EtOH) has the capacity to alter chromatin structure, which suggests that epigenetic mechanisms may be relevant to the genesis of birth defects associated with fetal alcohol spectrum disorders (FASDs) [2–4]. For example, studies examining tissue samples derived from both humans and rodents chronically exposed to alcohol have shown alterations in the levels of both the DNA methylating enzyme DNA methyltransferase 1 (Dnmt1) and DNA methylation within the regulatory regions of multiple genes; including those regulated though genomic imprinting [5–9]. In addition, multiple in vitro studies have revealed alterations in post-translational histone modifications arising as a consequence of ethanol exposure [10–14]. However, important questions as to the lasting heritability, the mechanism of induction, and the role these epigenetic errors have in the development of FASD-associated congenital malformations remain to be resolved.

In adults, alcohol is converted to acetaldehyde in an oxidation reaction that occurs primarily in the liver, and is driven by the enzymes alcohol dehydrogenase (ADH), cytochrome P450 (CYP2E1), and catalase. Even under normal physiologic conditions, this process produces excess acetaldehyde, reactive oxygen species (ROS) and other harmful adducts inducing oxidative stress [15]. Due to the free passage of alcohol from mother to fetus, and the constant recycling of the amniotic fluid reservoir, fetal alcohol exposures achieve blood alcohol concentrations equivalent to the mother’s, but of longer durations [16–18]. These longer exposures produce significant levels of ROS and free radicals, which have been hypothesized to be a significant factor in the teratogenic effects of alcohol [15, 19, 20]. Recently, a link between oxidative stress and the enzymes regulating chromatin structure has been identified [21]. These observations would suggest that some of the epigenetic changes induced by alcohol may be linked to alterations in the activities of genes responding to ROS [15, 20]. However, no study has yet directly examined interactions between alcohol exposures, oxidative stress and chromatin structure.

To date, the large majority of studies examining epigenetic changes arising from fetal alcohol exposures have employed either chronic models of constant exposure or examinations of acute alterations in chromatin structure [5–7, 10–12, 22–30]. Very few of these studies have sought to investigate the lasting heritability of EtOH-induced changes in chromatin structure arising from an acute encounter through development. This is significant as while there is ample evidence that chromatin structure can be perturbed by external factors, significant questions remain as to whether post-translational histone modifications are heritable, and can possibly contribute to environmentally induced phenotypes [31]. Are EtOH-induced alterations in histone modifications causal in FASD phenotypes, or are they merely transient differences between chromatin states induced by transcriptional/nucleosome remodeling responses to alcohol?

Using an ex vivo mouse model for fetal neural stem cells, our laboratory has shown dramatic reductions in histone 3 lysine 27 trimethylation (H3K27me3) in response to acute EtOH exposure, but no correlative alterations in the localization of the histone methyltransferase EZH2 were observed [10]. Current models of epigenetic inheritance suggest that during S-phase, chromatin modifying enzymes re-establish the histone code on newly assembled unmethylated histones, and therefore enzyme complexes, like the *Polycomb* group, represent the true locus-specific epigenetic mark passed from one generation to the next [32]. These observations therefore call into question the heritability of alcohol-induced epigenetic alterations, and their capacity to contribute to fetal alcohol syndrome (FAS) phenotypes. This is especially significant as chromatin modifications induced by exposures to other drugs of abuse tend to be transient, and revert back to control states within hours or days after the toxicant is removed [33].

In this study, we sought to examine two major questions: (1) are the epigenetic modifications induced by alcohol associated with a mobilization of epigenetic modifying genes downstream of the oxidative stress pathways, and (2) do alcohol-induced changes in chromatin structure persist beyond the window of exposure? We report multiple post-translational histone modifications display unique, dose-dependent responses to EtOH exposure, and in many cases, the epigenetic signatures arising after an acute exposure differ from those observed after a recovery period. These changes in chromatin structure are associated with persistent alterations in transcripts encoding *Dnmt1*, *Ehmt2* (*G9a*), *Eed*, *Ezh2*, *Kdm1a*, *Kdm4c*, *Setdb1*, *Sod3*, *Tet1* and *Uhrf1*. Transitioning into an in vivo model, we observe that mice displaying craniofacial malformations and midline brain defects arising from an acute, early gestational exposure also display epigenetic errors, and that these signatures of change are consistent with those modeled in vitro after a recovery period. Our results indicate that the immediate and long-term impacts of EtOH exposure on chromatin structure are distinct and suggest the existence of a coordinated cellular response to exposure. Importantly, an epigenetic signature resulting from an acute gestational encounter persists beyond the window of exposure, and strongly correlates with the appearance of congenital malformations.

## Results

### Acute and post-recovery epigenetic signatures of EtOH exposure display distinct, dose-dependent profiles

We sought to model the capacity of primary fetal cerebral cortical neuroepithelial stem cells to restore alcohol-induced alterations in chromatin structure. To this end, we initiated a treatment protocol wherein cells were maintained in the stem cell state and exposed to varying concentrations of EtOH for 3 days; then allowed to progress through a 4-day recovery period representing at least three population doublings. The concentrations of alcohol utilized in this study were meant to mimic those obtained from a binge drinker. We utilized projected concentrations based on observations by White et al., which demonstrated that out of 7356 college age females surveyed, 33.7 % reported typical consumption rates at 1× binge alcohol levels (four or more drinks at a time) and 8.2 % reported consumption at 2× binge levels (eight or more drinks at a time) [34]. Based on average height and weight, these rates of consumption would yield blood alcohol levels in the range of 160 and 240 mg/dL, respectively (blood alcohol content—http://www.dot.wisconsin.gov).

To measure the impact of EtOH exposure and withdrawal on chromatin structure, control and alcohol-treated cellular extracts were immunoprecipitated with antibodies recognizing the specific chromatin modifications trimethylated histone 3 lysine 4 (H3K4 me3), trimethylated histone 3 lysine 27 (H3K27 me3), acetylated histone 3 lysine 9 (H3K9 ac) and dimethylated histone 3 lysine 9 (H3K9 me2) using methods previously utilized by our laboratory [35]. Alterations of these histone post-translational modifications have been observed in previous studies of acute alcohol exposure, but their capacity to restore over time has not been investigated [10–14]. To examine gene-specific alcohol-induced changes in chromatin structure, DNA fragments isolated from these precipitations were examined by qPCR relative to 1 % of the total input. Using primers homologous to sequences ±250 base pairs of the transcriptional start sites, we assessed alterations in chromatin structure occurring within the regulatory regions of 22 genes randomly distributed across the genome that are involved in controlling multiple growth factor signaling pathways directing neuronal patterning. Importantly, we and others have identified altered transcriptional control and aberrant localization of transcripts encoding many of these genes in FASD mouse models, thus making them suitable candidates for the current study [36–39].

**Fig. 1:**
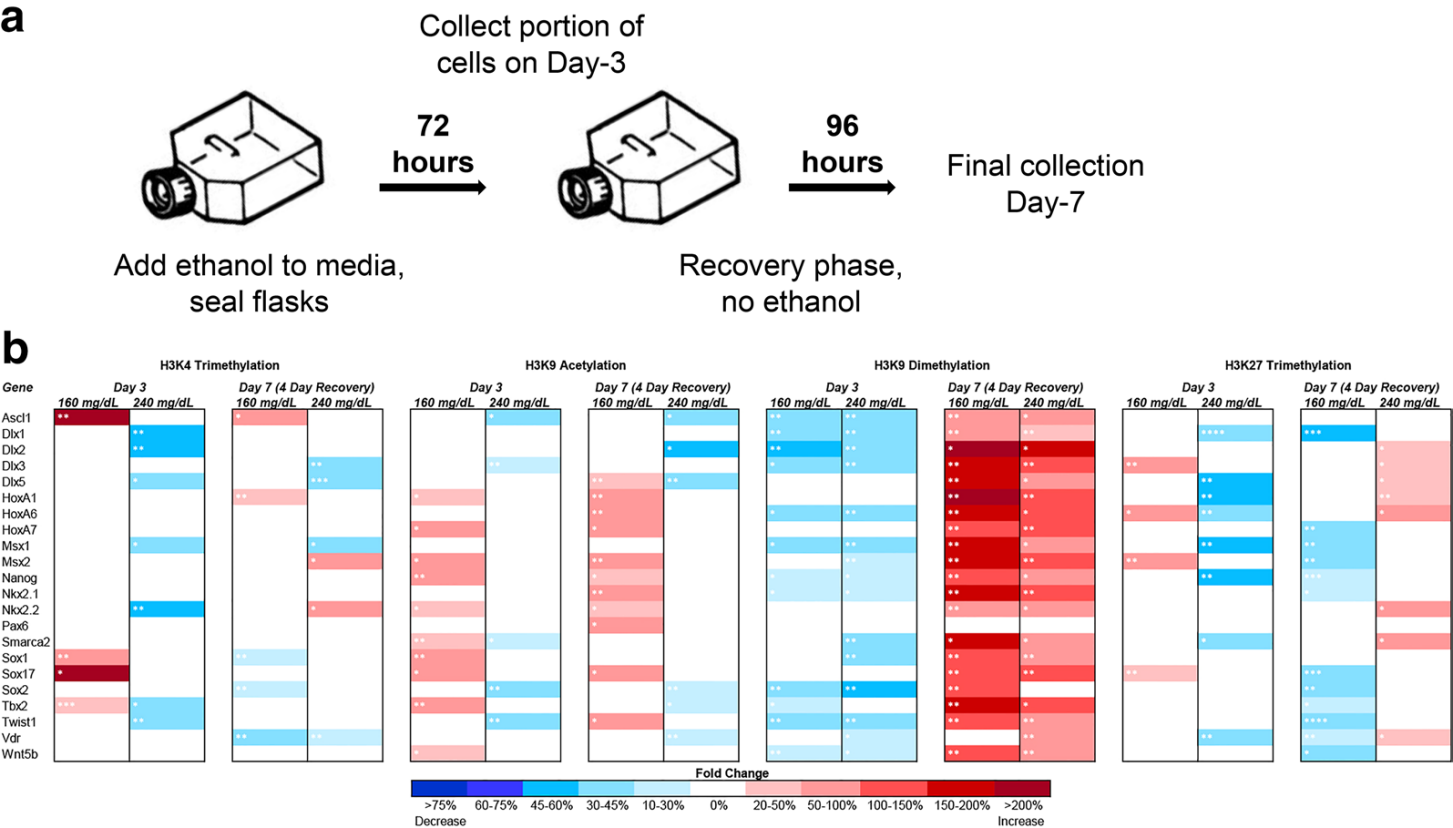
Dose-dependent epigenetic signatures of EtOH exposure persist past the period of exposure. **a** Experimental paradigm. **b** Alcohol-induced epigenetic alterations in H3K4 me3, H3K9 ac, H3K9 me2, and H3K27 me3. Primary fetal cerebral cortical neuroepithelial stem cells were cultured in the presence of 160 or 240 mg/dL EtOH for 3 days, followed by a 4-day recovery in medium lacking EtOH. Samples were collected at days 3 and 7, and examined for changes in the indicated post-translational histone modifications using chromatin immunoprecipitation followed by quantitative PCR (ChIP-qPCR). Heat maps represent fold change in H3K4 me3, H3K9 ac, H3K9 me2, and H3K27 me3 within the regulatory regions of the genes listed. Primers were designed to fall within 250 base pairs of the transcriptional start site. Within the three separate biological replicates (*N* = 3), three ChIPs were performed, and two qPCR replicates performed on each independent ChIP. Statistical measures were conducted using the Wilcoxon signed rank nonparametric test. *p < 0.05; **p < 0.01; ***p < 0.001; ****p < 0.0001

The comprehensive results of these independent analyses are presented as a heat map in Fig. 1, with the statistical significance demarcated in each cell. Analysis of individual candidate genes may be viewed in Additional file 1. For the post-translational marks examined, we observed a range of alterations on the order of 45 % reductions to more than 200 % increases. These scales of change are similar to those reported in experiments utilizing either over-expression or RNA interference mediated suppression of key epigenetic modifiers [EHMT1/G9a and Polycomb Repressive Complex 2 (*PRC2*)] [40–43]. Collectively, these experiments produced five novel observations. First, the examined histone post-translational modifications were not equally impacted by alcohol exposure. Broadly, the candidate genes examined displayed modest alterations in H3K4 me3, more pronounced changes in H3K27 me3 and H3K9 ac, and profound shifts in H3K9 me2 across all treatment groups and time points examined. It is interesting to note that epigenetic modifications associated with a condensed chromatin architecture displayed the largest and most frequent changes.

Second, we observed that a loss of histone marks associated with repressive chromatin structure did not immediately correlate with gains in post-translational modifications associated with relaxed chromatin structure, and similarly, loci displaying decreases in marks associated with transcription did not see increases in post-translational modifications associated with gene repression. A number of studies have suggested an interdependent relationship between many of the marks examined here, yet even after a recovery period, this lack of correlation persists. Third, a clear dose-dependent effect exists between the epigenetic changes observed in the 160 mg/dL and the 240 mg/dL treatment groups. While the 160 mg/dL treatment elicited an enrichment of H3K4 me3, H3K9 ac, and H3K27 me3, depletion of these histone marks were observed in the 240 mg/dL treatment groups. Only H3K9 me2 exhibited a uniform depletion across treatments at this time point.

Fourth, when extracts obtained after a 4-day recovery were examined (Day-7), we noticed a persisting signature of exposure that was unique to each post-translational modification examined. For example, gene promoters displaying alterations in chromatin modifications associated with relaxed chromatin (H3K9 ac and to a lesser extent H3K4 me3) on Day-3, maintained the observed altered profiles on Day-7. In contrast, loci that had become depleted for marks associated with compacted chromatin (H3K27 me3, and H3K9 me2) on Day-3 displayed a hypermethylated state on Day-7, suggesting these genes had been remodeled into a repressive chromatin state. The exception being the H3K27 me3 in the 160 mg/dL treatments, which were hypermethylated on Day-3 and became depleted on Day-7. In addition, we observed that many genes displaying chromatin profiles identical to the control on Day-3 exhibited altered profiles on Day-7 and several alterations present on Day-3 resolved at the Day-7 time point. As examples, the regulatory regions of *Dlx5* and *Nkx2.2* displayed H3K9 me2 and H3K27 me3 profiles identical to the control on Day-3, but became hypermethylated by Day-7. Finally, not all genes were uniformly affected in our system, and many only displayed alterations in a subset of the post-translational modifications examined. The regulatory region of *Pax6* for instance only exhibited changes in H3K9ac at the Day-7 time point, while all other chromatin marks were identical to the controls, at the time points examined. In contrast, *Ascl1*, *Msx2*, *Nkx2.2* and *Tbx2* all displayed significant changes in at least three of the four histone marks examined, and these changes varied across the range of concentrations tested and time points examined. Collectively, these results suggest that the epigenetic changes arising as a consequence of EtOH exposure are heavily dependent on the gene under investigation, the dose of alcohol encountered, the epigenetic mark under investigation, and that the profile of change arising acutely is not always consistent with ones measured after removal of the toxicant. These observations may have relevance to understanding the molecular basis underlying the enormous variation observed in clinical cases of FASDs.

### EtOH exposure in vitro is associated with alterations in transcripts encoding *Sod3* and *Tet1*, but no alterations in markers of cell death, oxidative stress, nor significant disruption of the oxidative stress transcriptional response

**Fig. 2:**
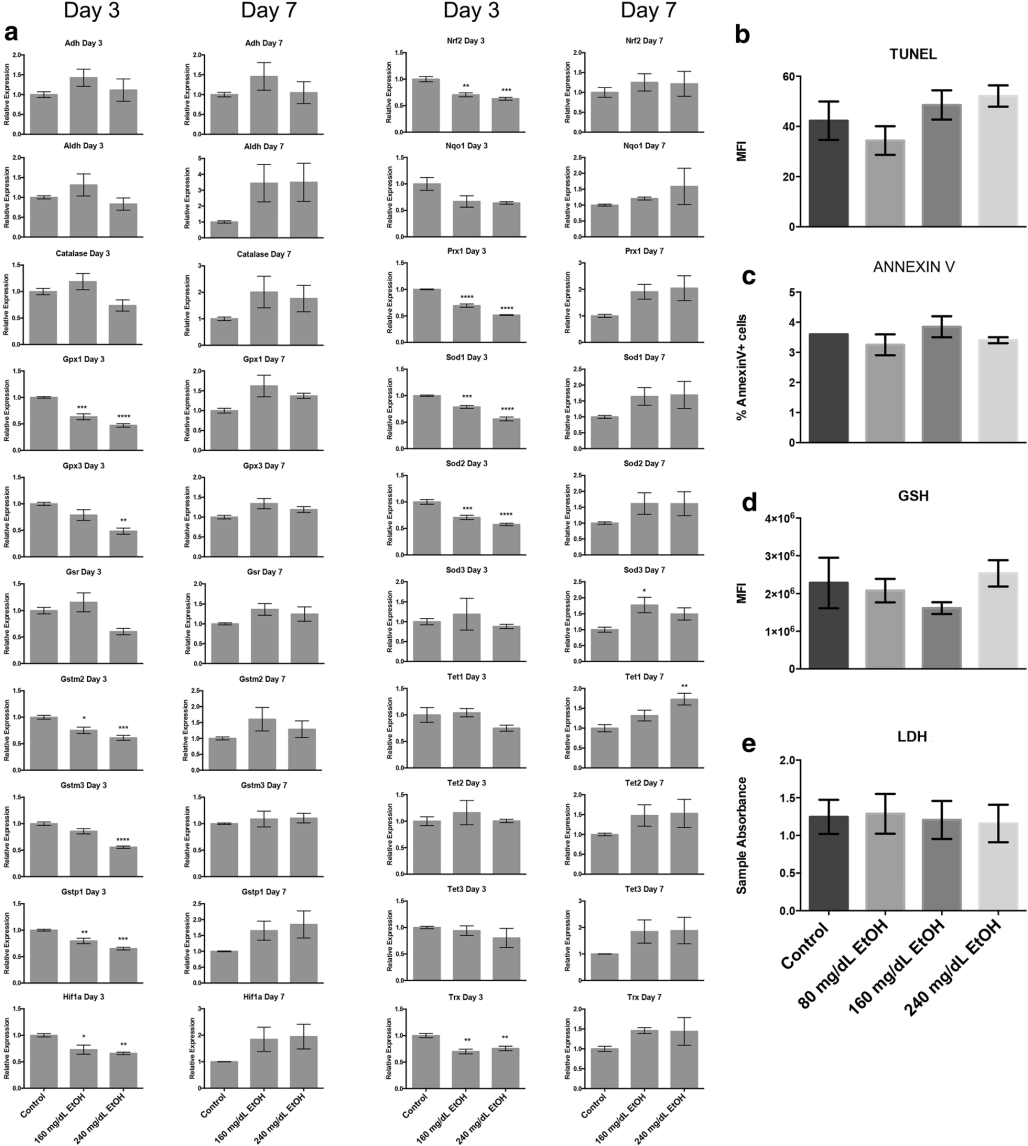
EtOH exposure in vitro alters levels of transcripts encoding *Sod3* and *Tet1*, but does not impact measures of cell death or oxidative stress. **a** Measurements of transcripts encoding proteins involved in the metabolic processing of alcohol and oxidative stress response pathways. Primary neuroepithelial stem cells were cultured in the presence of 160 or 240 mg/dL EtOH for 3 days, followed by a 4-day recovery in media lacking EtOH. Samples were harvested at days-3 and 7, and transcript levels determined by RT-qPCR. *Graphs* represent three independent biological replicates (*N* = 3), with two independent RT reactions and three independent qPCR measurements for each RT. Significance was measured using a one-way ANOVA, *error bars* represent SEM. *p < 0.05; **p < 0.01; ***p < 0.001; ****p < 0.0001. **b**–**e** Measures of cellular stress and apoptosis in primary neuroepithelial stem cells exposed to alcohol. Cells were cultured in the presence of 80–240 mg/dL EtOH for 3 days, then assayed for markers of **b** and **c** apoptosis, **d** oxidative stress and **e** cellular stress. Differences were measured using a one-way ANOVA, *error bars* represent SEM. *Graphs* represent three separate biological replicates (*N* = 3)

Given the observed increases in ROS following alcohol exposure, researchers have speculated that some of the teratogenicity associated with EtOH exposure is linked to oxidative stress [15, 44]. Recently, a link between components of the oxidative stress pathways and enzymes controlling chromatin structure has been identified [21, 45]. To examine a potential link between mobilization of the oxidative stress response, and the observed alterations in chromatin structure, we began by quantifying the transcript levels of 23 well characterized candidate genes involved in either the metabolic processing of alcohol or the oxidative stress response pathway [44]. Of these 23 candidates, transcripts encoding *Cyp2e1*, *Gpx2* and *Gsta2* could not be detected in RNA samples isolated from our neurosphere cultures. Surprisingly, of the remaining 20 candidates, the majority of genes exhibited a down-regulation at the Day-3 time point and no significant alterations at Day-7 (Fig. 2a). The two notable exceptions to this trend were *Sod3*, and *Tet1*, which measured a 1.7-fold increase over the control at the Day-7 time point.

We next measured physiological parameters associated with apoptosis, oxidative stress and cytotoxicity. Neurosphere cultures were treated with 80, 160, and 240 mg/dL EtOH for 72 h, and subsequently examined using Annexin 5 and TUNEL assays (Fig. 2b, c). Neither of these tests indicated a significant increase in levels of cellular apoptosis. When we examined our treatment groups for alterations in the levels of glutathione (GSH), decreases of which are associated with oxidative stress, no significant changes were observed (Fig. 2d). We next examined cell cultures for increased levels of lactate dehydrogenase (LDH), a marker of generic cell stress. Again, this parameter did not show any significant changes across the range of EtOH concentrations examined (Fig. 2e). These observations suggest that the oxidative stress pathways are not broadly engaged in our in vitro model of fetal alcohol exposure; at least not at the concentrations examined.

### EtOH exposure in vitro is not associated with an inhibition of histone methyltransferase enzymatic activity but does induce alterations in transcriptional regulation

**Fig. 3:**
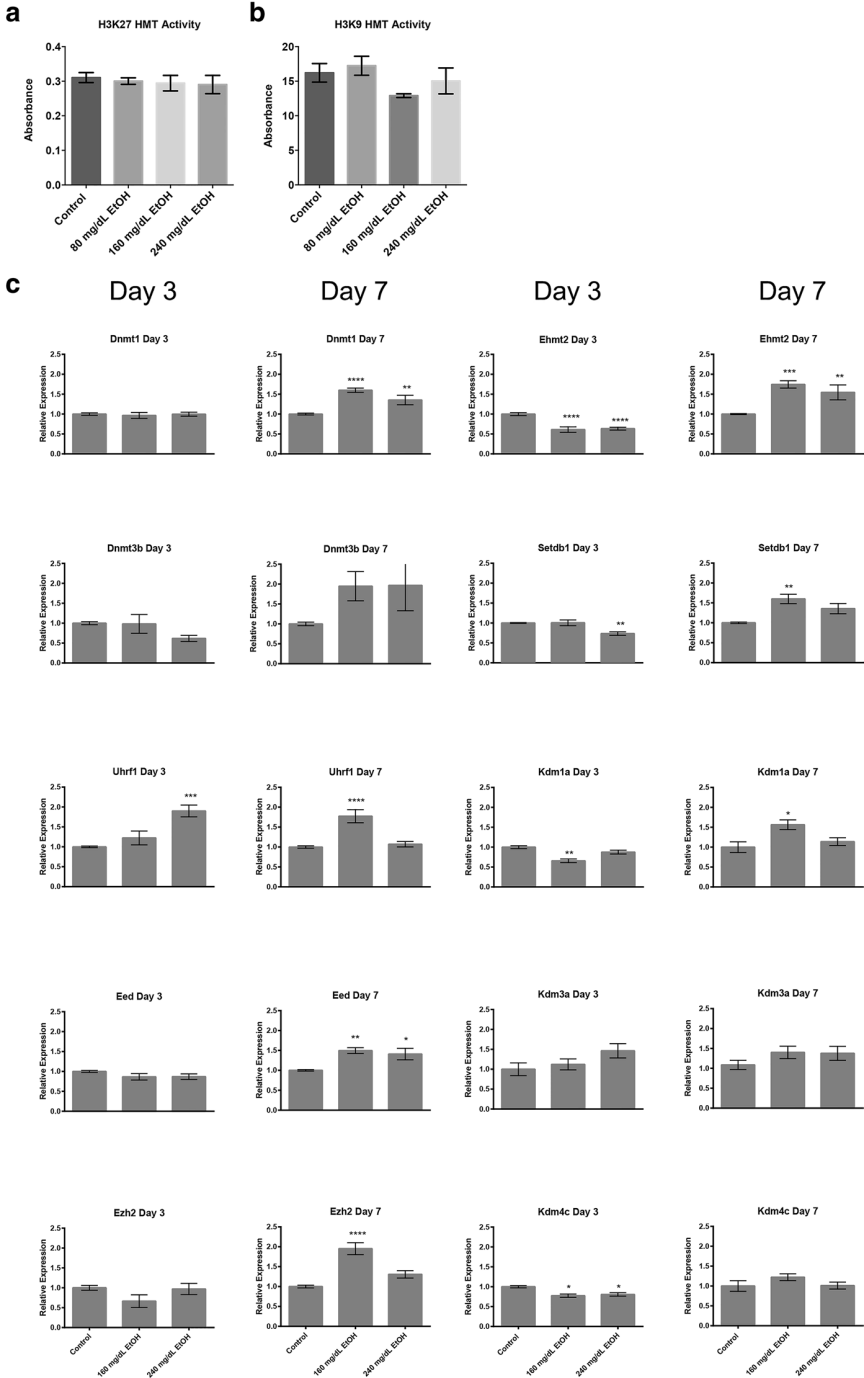
In vitro EtOH exposure does not inhibit methyltransferase enzymatic activity, but does induce alterations in the transcriptional control of DNA and Histone methyltransferase enzymes. **a**, **b** Measures of histone methyltransferase activity in EtOH-exposed neuroepithelial stem cells. Cells were cultured in the presence of 80–240 mg/dL EtOH for 3 days, and cellular extracts assayed for **a** H3K27 and **b** H3K9 histone methyltransferase activity using a colorimetric assay. Differences were measured using a one-way ANOVA. *Error bars* represent SEM. *N* = 3. **c** Measurement of transcripts encoding enzymes governing DNA, H3K9, and H3K27 methylation. Primary neuroepithelial stem cells were cultured in the presence of 160 or 240 mg/dL EtOH for 3 days, followed by a 4-day recovery in media lacking EtOH. Samples were harvested at days-3 and 7, and transcript levels determined by RT-qPCR. *Graphs* represent three independent biological replicates (*N* = 3), with two independent RT reactions and three qPCR measurements for each RT. Significance was measured using a one-way ANOVA, *error bars* represent SEM. *p < 0.05; **p < 0.01; ***p < 0.001; ****p < 0.0001

Work by our group has demonstrated reductions in H3K27me3 arising due to acute EtOH exposure [10]. We therefore sought to determine if the acute losses of H3K9 me2 and H3K27 me3 observed in our cell culture model could possibly be linked to alcohol-induced inhibition of histone methyltransferase enzyme activity. To this end, cell cultures were treated with an EtOH dose response range from 80 to 240 mg/dL, and both H3K9 and H3K27 methyltransferase activity quantified using a colorimetric assay. Neither significant differences in H3K9 nor H3K27 methyltransferase activity were observed across the range of concentrations tested (Fig. 3a, b).

We then examined levels of transcripts encoding ten major proteins responsible for regulating DNA methylation as well as H3K9 and H3K27 methylation (Fig. 3c) [46–55]. Interestingly, transcripts encoding *Ehmt2* (*G9a*) and *Setdb1*, the two enzymes responsible for methylating H3K9, display alcohol-induced suppression on Day-3 and an up-regulation on Day-7; which is consistent with the observations in Fig. 1. However, on Day-3, we also observed decreases in the abundance of transcripts encoding *Kdm1a*, and *Kdm4c*, as well as a modest increase in *Kdm1a* on Day-7. These two enzymes have established roles in demethylating H3K9 [54, 55]. None of the other factors examined display altered transcript profiles on Day-3, with the exception of *Uhrf1* in the 240 mg/dL treatments. In contrast, *Dnmt1*, *Uhrf1*, *Eed*, and *Ezh2* all exhibited alterations on Day-7. These observations suggest some of the alterations in chromatin structure may be tied to changes in the levels of enzymes regulating DNA/histone methylation.

### EtOH-induced alterations in *Dnmt1*, *Tet1* and *Uhrf1* transcript levels are associated with measurable alterations in DNA methylation but not DNA hydroxymethylation

Recently, it has been shown that the TET family of Fe(II) and α-KG-dependent dioxygenases rely upon oxygen to convert 5-methyl-cytosine (5mC) into 5-hydroxy-methyl-cytosine (5hmC) [56]. This modified form of cytosine is abundant in the brain and is hypothesized to play a key role in the epigenetic control of neuronal function [57]. Importantly, the formation of 5hmC can lead to demethylation of DNA, which in turn can influence other aspects of chromatin structure; including H3K4 me3, H3K9 me2, and H3K27 me3 [58–60]. Since our transcript profiles, as well as previous studies in other models [8, 61] have identified alterations in gene family members regulating both 5mC and 5hmC, we set out to determine these alterations were associated with gene-specific changes in DNA methylation/hydroxy methylation. To this end, we utilized glucosylation of genomic DNA followed by methylation-sensitive qPCR (glucMS-qPCR) to examine alcohol-induced alterations in eight candidate genes. These candidates were identified in previous studies of 5hmC within the brain and embryonic stem cell-derived neural progenitor cells [62–64].

**Fig. 4:**
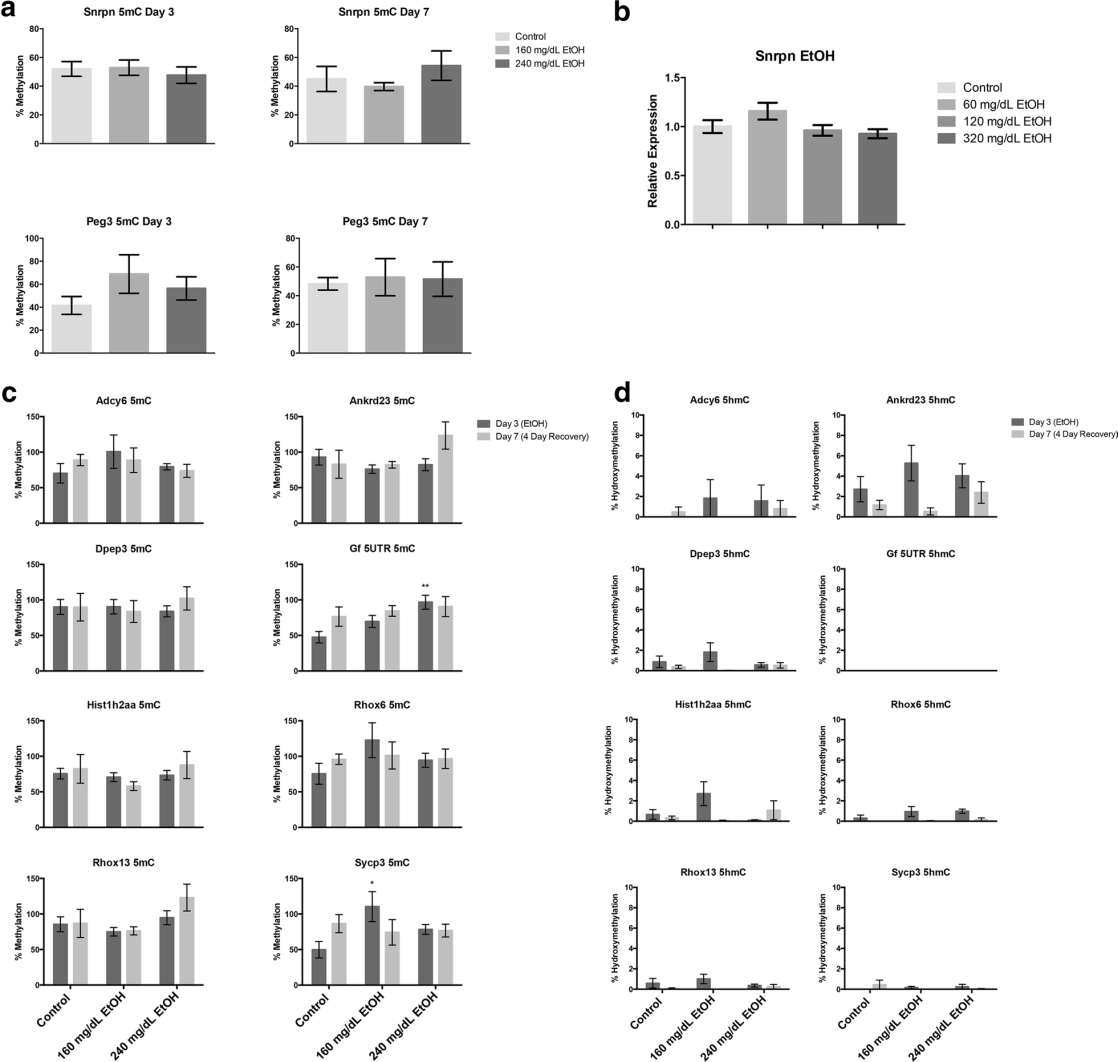
Alterations in DNA methylation but not hydroxymethylation in EtOH-exposed primary neuroepithelial stem cell cultures. **a** Stable levels of DNA methylation within the differentially methylated regions of the imprinted genes *Snrpn* and *Peg3*. Stem cells were cultured in the presence of 160 or 240 mg/dL EtOH for 3 days, followed by a 4-day recovery in media lacking EtOH. Genomic DNA was collected at days-3 and 7, and analyzed for alterations in DNA 5mC and 5hmC using glucMS-qPCR. *Graphs* represent three independent biological replicates (*N* = 3), with three qPCR measurements each. **b** Quantification of Snrpn transcript levels using RT-qPCR. *Graphs* represent three independent replicates (*N* = 3), with two independent RT reactions and three qPCR measurements for each RT. Differences were measured using a one-way ANOVA, *error bars* represent SEM. **c**, **d** Measurement of 5mC and 5hmC within the regulatory regions of eight genes identified in previous studies of DNA hydroxymethylation. Cells were cultured in the presence of 160 or 240 mg/dL EtOH for 3 days, followed by a 4-day recovery in media lacking EtOH. Genomic DNA was collected at days-3 and 7, and analyzed for alterations in DNA **c** 5mC and **d** 5hmC using glucMS-qPCR. Differences were measured using a one-way ANOVA, *error bars* represent SEM. *Graphs* represent three independent biological replicates (*N* = 3), with three qPCR measurements each

To first validate our methodologies, we examined levels of 5mC within the differentially methylated regions of two imprinted genes (*Peg3* and *Snrpn*) [65]. Both candidates demonstrated 50 % 5mC consistent with one allele being methylated and the other unmodified; but no detectable 5hmC (Fig. 4a). At these two loci, no significant alterations in 5mC were induced by alcohol exposure across the range of concentrations tested and time points examined. We then evaluated expression of *Snrpn* in EtOH-exposed cultures and did not observe any significant changes, consistent with the stable measures of 5mC at this locus (Fig. 4b). We next assayed alterations in both 5mC and 5hmC within either the gene bodies or regulatory regions of eight candidate genes identified in previous studies of 5hmC. We observed increases in 5mC within the 5′UTR of *Gf* and the regulatory region of *Sycp3* (Fig. 4c). While we were able to detect very low levels of 5hmC consistent with previous reports [62–64], none of these loci exhibited alcohol-induced changes as compared to the controls (Fig. 4d). In our primary cultures, the 5′UTR of *Gf* did not exhibit any detectable 5hmc. Collectively, these results suggest that while the observed increase in DNA methyltransferase levels are correlated with modest increases in 5mC, increased *Tet1* transcript levels are not associated with any measurable changes in 5hmC across the candidate loci examined.

### Alterations in *Homeobox* gene transcription predominantly manifest beyond the window of EtOH exposure

**Fig. 5:**
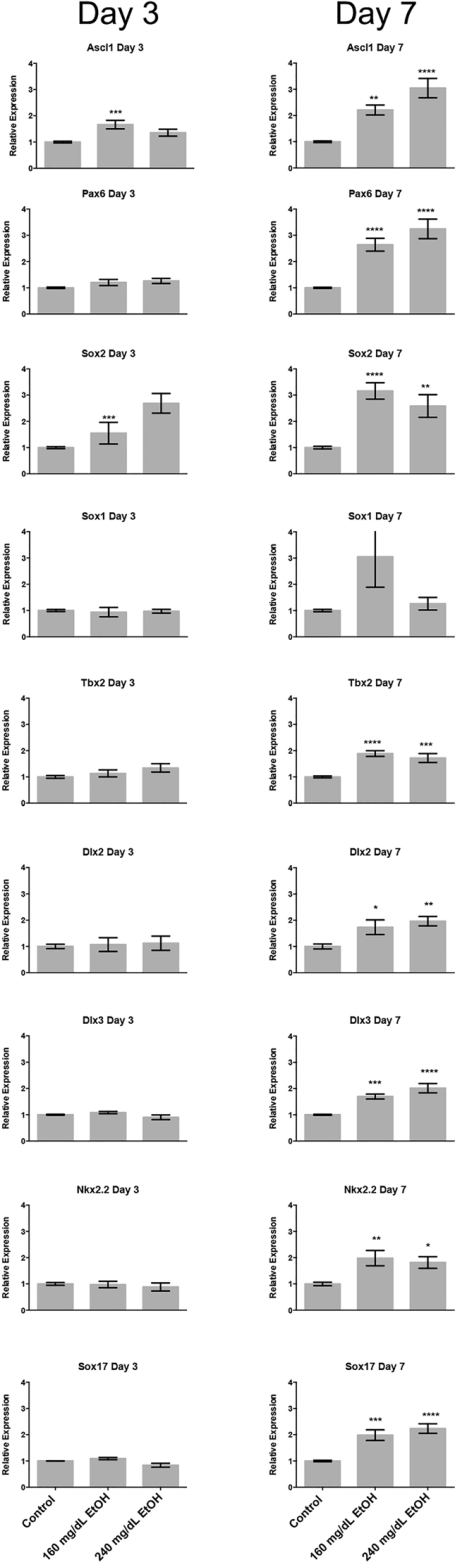
Distinct alterations in *homeobox* gene transcription arising during and after the window of EtOH exposure. Neural stem cells were cultured in the presence of 160 or 240 mg/dL EtOH for 3 days, followed by a 4-day recovery in media lacking EtOH. Cells were harvested at days-3 and 7, and transcript levels measured using RT-qPCR. *Graphs* represent three independent replicates (*N* = 3), with two independent RT reactions and three qPCR measurements for each RT. Differences were measured using a one-way ANOVA, *error bars* represent SEM. *p < 0.05; **p < 0.01; ***p < 0.001; ****p < 0.0001

**Fig. 6:**
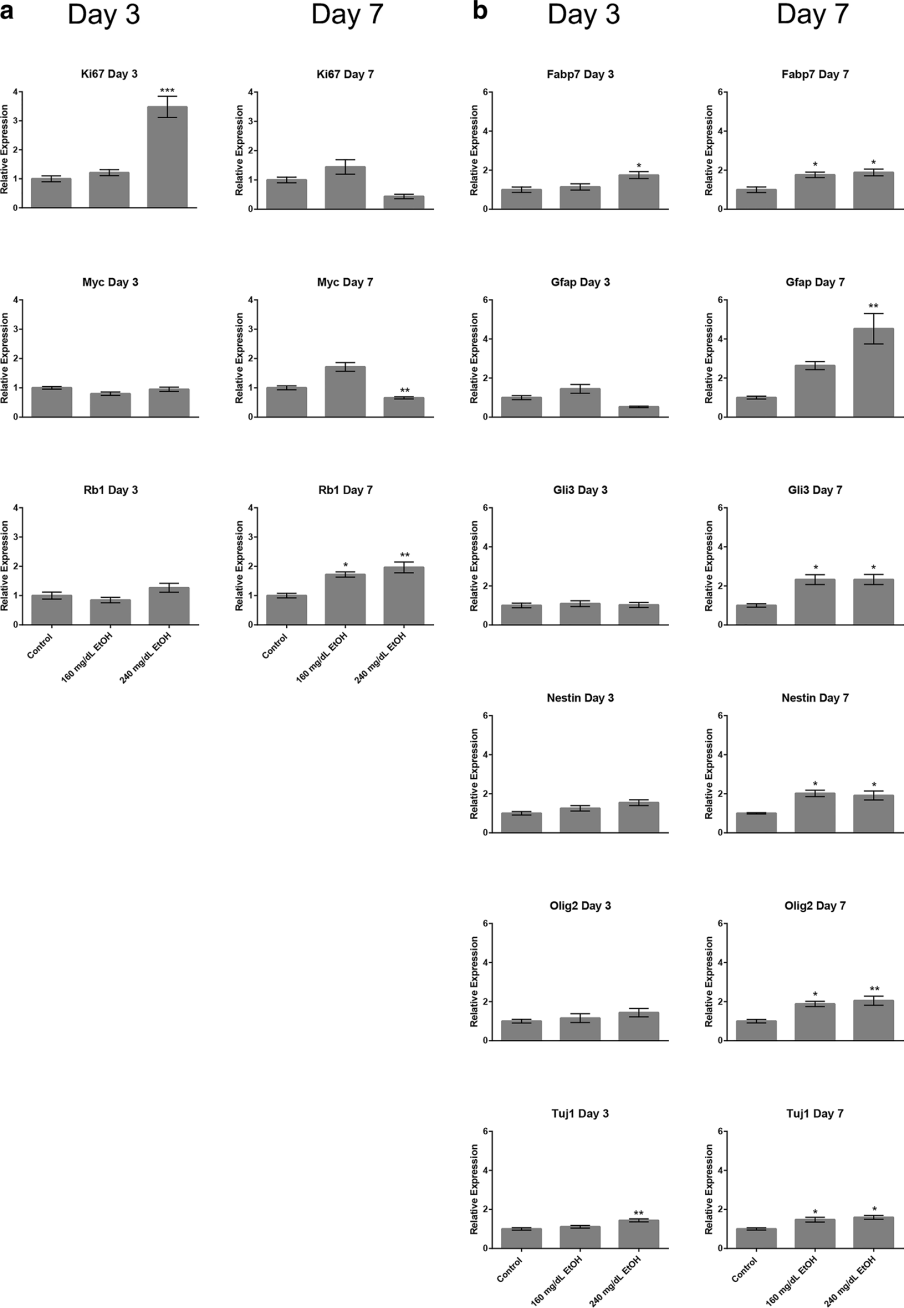
Alterations in transcripts encoding proteins regulating neural stem cell identity and proliferation predominantly arise after the window of EtOH exposure. Neural stem cells were cultured in the presence of 160 or 240 mg/dL EtOH for 3 days, followed by a 4-day recovery in media lacking EtOH. Cells were harvested at days-3 and 7, and transcript levels measured using RT-qPCR. *Graphs* represent three independent replicates (*N* = 3), with two independent RT reactions and three qPCR measurements for each RT. Differences were measured using a one-way ANOVA, *error bars* represent SEM. *p < 0.05; **p < 0.01; ***p < 0.001

Published reports examining acute EtOH exposure have been unable to demonstrate consistent correlations between alterations in H3K4 me3; a histone mark enriched at the promoter regions of actively transcribed genes [66], and changes in transcription [10, 67, 68]. We were therefore curious to determine if the candidate genes demonstrating changes in any of the measured chromatin marks either before or after the recovery period would display alterations in gene transcription. To this end, RNA was isolated from all treatment groups and gene expression measured using quantitative reverse transcriptase polymerase chain reaction (qRT-PCR). Of the 22 candidate genes examined in Fig. 1, transcripts encoding *Ascl1*, *Dlx2*, *Dlx3*, *Pax6*, *Nkx2.2*, *Sox1*, *Sox2*, *Sox17*, and *Tbx2* could be detected in our cultures (Fig. 5). Similar to our previous studies [10], only a very small number (20 %) of candidate genes (*Ascl1* and *Sox2*) displayed altered expression profiles arising as a consequence of EtOH exposure at Day-3. In contrast, eight of the nine detected candidates (~88 %) displayed significant alterations in transcript levels on Day-7, across both concentrations of EtOH tested. These candidate homeobox genes sit at the hub of multiple transcriptional pathways controlling cellular identity and proliferation. We therefore assayed RNA samples for alterations in the expression of known markers of both cellular growth (*Ki67*, *cMyc*, and *Rb1*) and neural stem cell proliferation/identity (*Fabp7*, *Gfap*, *Gli3*, *Nestin*, *Olig2* and *Tuj1*). These analyses revealed changes in a small number of candidates within the 240 mg/dL treatments on Day-3 (*Ki67*, *Fabp7*, and *Tuj1*), while the larger impact was again observed on Day-7, with eight of the nine candidates demonstrating alterations in transcription (Fig. 6). These observations indicate the larger impact of EtOH exposure on the developmental program may arise beyond the initial period of exposure.

### An epigenetic signature of EtOH exposure arising from an acute gestational encounter persists beyond the window of exposure

Abnormalities in the cortex of the brain are often associated with alcohol-induced impairments in high-level sensory and motor processing, as well as with some FASD cognitive-behavioral phenotypes. As our stem cell cultures are derived from mouse cerebral cortex precursors, we sought to assess the relevance of our in vitro observations on the development of FASD-associated birth defects. The C57Bl/6J mouse has been critical in defining some of the stage-dependent dysmorphologies resulting from acute early gestational ethanol exposures. An acute binge-like ethanol treatment on gestational day 7 (GD7) (equivalent to the early third week of human development—gastrulation) results in a range of grossly observable fetal anomalies such as holoprosencephaly and classic FAS facial characteristics [69, 70]. Importantly, both the craniofacial and midline brain anomalies can be consistently scored and their degree of severity correlated with concurrently developing defects within the CNS [70, 71]. We therefore examined the prevalence of altered chromatin structure within the cortex of mouse pups exposed to an early, binge-like gestational exposure.

**Fig. 7:**
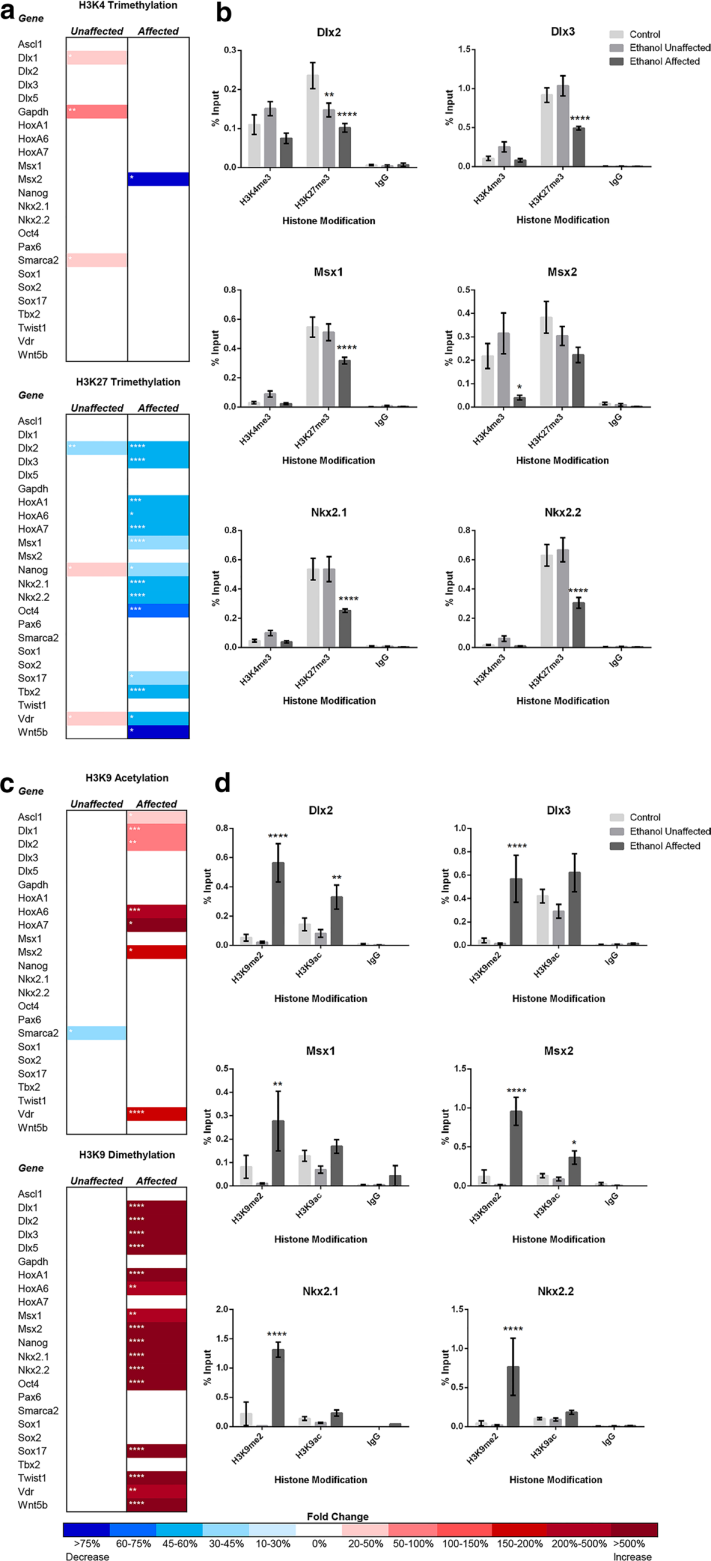
Lasting alcohol-induced alterations in H3K4 me3, H3K9 ac, H3K9 me2, and H3K27 me3 within the prenatal cortex arising from an early gestational exposure. Pregnant dams were injected with 2.9 g/kg EtOH at GD7 and embryos harvested at GD17. Embryos were scored for ocular and cortical patterning defects, and sorted into three groups—control, EtOH exposed—morphologically normal, and EtOH exposed—malformed. After dissection of the fetal cortex, ChIP-qPCR analysis was performed on cellular extracts using antibodies recognizing H3K4 me3, H3K9 ac, H3K9 me2, and H3K27me3. **a**, **c** Heat maps representing fold change in the levels of the indicated post-translational modifications relative to samples derived from saline-exposed controls. In the experiments examining H3K4 me3 and H3K27 me3, four ChIP experiments were performed on a total of 15 brains across 5 different litters. For analysis of H3K9 ac and H3K9 me2, three ChIP experiments were performed on a total of 10 brains across 5 different litters. Two replicates of qPCR were performed on each ChIP. Significance was determined using a two-way ANOVA. *Error bars* represent SEM.*p < 0.05; **p < 0.01; ***p < 0.001; ****p < 0.0001. **b**, **d** Representative *graphs* depicting alcohol-induced alterations in chromatin structure. A complete analysis of individual genes may be viewed in Additional file 2

On GD7, pregnant dams were intraperitoneally administered two injections of either vehicle or 2.9 g/kg EtOH 4 h apart, yielding peak maternal blood alcohol concentrations averaging 440 mg/dL [70]. These blood alcohol concentrations are much higher than those utilized in our in vitro studies; however, in mouse models of FAS, lower concentrations do not consistently produce holoprosencephaly and classic FAS facial characteristics [71]. We therefore elected to use a treatment paradigm that would produce a low, but consistent frequency of alcohol-induced birth defects, yet that was not overtly toxic. On GD17, stage-matched control and EtOH-exposed fetuses were dissected, and scored for ocular defects as described previously [71]. Using this model, 12 % of the EtOH-exposed pups displayed holoprosencephaly and FAS facial characteristics, whereas the remaining animals were morphologically normal. Mice were then sorted into groups by treatment and morphological appearance. In total, 25 mice from 6 different litters were selected for analysis: 10 control, 8 EtOH exposed—morphologically normal, and 7 EtOH exposed—malformed. To examine the impact of EtOH exposure upon the epigenetic program of the developing central nervous system, the fetal cortex was dissected out, and using chromatin immunoprecipitation, we assayed cellular extracts for alterations in the chromatin marks examined above (Fig. 7).

In samples derived from EtOH exposed—malformed pups, we observed a pattern of change that largely correlated with the in vitro post-recovery signature observed in the 160 mg/dL treatment group in Fig. 1. Fetal mice exhibiting craniofacial dysmorphology and midline brain defects displayed loss of H3K27 me3 in 14 of the 24 (58 %) candidates examined, a modest enrichment of H3K9 ac in 7/24 candidate genes (29 %), and a dramatic increase in H3K9 me2 in 17/24 (70 %) of the candidate regulatory regions examined. The hypermethylated state of H3K9 in particular was robust and very consistent. With the exception of a modest change within the promoter region of *Msx2*, no alterations of H3K4 me3 were observed in the EtOH exposed—malformed tissue samples. It is interesting to note that across at least three of the four post-translational modifications examined, *Dlx2*, *HoxA6*, *HoxA7*, *Msx2*, and *Vdr* consistently displayed alterations in chromatin structure, suggesting certain genes may be more susceptible to epigenetic errors than others. In our analysis, we noticed that post-translational modifications associated with repressive chromatin structure are profoundly impacted as compared to those associated with transcriptionally active, yet these changes move in opposite directions; H3K27 me3 is lost while H3K9 me2 is gained. This was surprising given EHMT2 (G9a) and the *Polycomb* group have been observed as part of a common complex, and on at least a subset of genes in ES cells, increased H3K9 me2 enhanced EZH2 activity [40]. In contrast, EtOH exposed—morphologically normal pups displayed modest changes in H3K4 me3 and H3K27 me3, while levels of H3K9 me2 were identical to those measured in the controls. Collectively, these observations indicate that an epigenetic signature of EtOH exposure persists beyond the window of exposure, and is largely linked to alterations in repressive chromatin structure; H3K9 me2 in particular. Importantly, these observed alterations strongly correlate with the development of FASD-associated congenital malformations.

## Discussion

Epidemiologic studies have shown that alcohol is the most prevalent teratogen to which humans are exposed, and in the United States, 6–9 infants per 1000 live births are diagnosed with some degree of fetal alcohol spectrum disorder [72]. Despite years of intense study, determining the developmental basis for the enormous variation in both the severity and range of birth defects linked to prenatal alcohol exposure remains a formidable challenge. We and others have demonstrated multiple alterations in chromatin structure arising as a consequence of EtOH exposure, but linking these epigenetic changes to alterations in the developmental program, and ultimately to the acquisition of congenital abnormalities, has yet to be achieved [2, 3]. Our observation that the two concentrations of EtOH tested in vitro were able to elicit distinct changes in chromatin structure suggest some of the variation in FAS clinical cases may be attributable to dose-dependent alterations in the epigenetic program. It is noteworthy that the direction of change arising from the 160 mg/dL exposure for H3K4 me3, H3K27 me3, and H3K9 ac are opposite to those induced by the 240 mg/dL treatments. Only H3K9 me2 was uniformly affected. The mechanisms underlying the observed non-linear dose responses in fetal alcohol exposure are unclear. However, such data emphasize that the teratogenic potential of ethanol is not diminished with lower doses.

Alterations in H3K9 me2 are consistently induced by EtOH exposure, persist beyond the window of exposure, and have a strong association with the development of congenital abnormalities. Previous studies of acute EtOH-induced liver injury, neuroplasticity, neurodegeneration, and neuroadaptation have also observed gene-specific changes in H3K9 methylation indicating alterations to this post-translational mark may be a core aspect of EtOH teratogenicity [11, 13, 73, 74]. The correlative shifts in transcript abundance of the major genes regulating the dynamics of H3K9 methylation support this assertion. Recently, two independent studies in yeast have reported a potential reader–writer mechanism of epigenetic inheritance for H3K9 methyl-marks [75, 76]. These observations suggest disruptions in H3K9 methylation may be heritable through development, and therefore represent a plausible mechanism of transmitting a lasting signature of EtOH exposure. If alterations at this residue are indeed linked to gestational EtOH exposures causing birth defects, we speculate this signature may be identifiable in clinical samples such as cord blood, and thus potentially serve as a biomarker of exposures linked to patterning defects. If true, this could yield the potential to identify FASD cases that do not present with overt craniofacial abnormalities, but yet have associated neurological deficits [72]. The predictive value of epigenomic markers over genetic ones is starting to gain wider acceptance [77], thus this strategy may be helpful in the delivery of FASD educational interventions at the earliest possible points.

Researchers have speculated that some of the teratogenicity associated with EtOH exposure is linked to oxidative stress, and inhibition of aspects of one carbon metabolism [15, 78]. Our data both in vitro and in vivo, suggest that some loci gain methylation while others exhibit a decrease. Specifically, the dramatic increases in H3K9 me3 observed within our candidate gene regulatory regions does not support the notion that a shortage of methyl groups underlies the observed changes in chromatin structure. However, our analysis is focused on select regulatory sequences and does not examine global changes in any of these post-translational histone marks, nor does it examine alterations in global levels of DNA methylation, which previous reports have found to be significantly reduced in EtOH-exposed animals [5, 8]. Additionally, we did not observe a correlation between epigenetic alterations brought on by in vitro EtOH exposure and changes in the examined markers of cell death, cell stress or oxidative stress. Although we did observe alterations in *Tet1* transcript levels, these were not associated with measurable changes in 5hmC. Thus, we were unable to find evidence supporting the notion that epigenetic modifications induced by alcohol are associated with genes downstream of the oxidative stress pathways, at least at the concentrations examined here.

Current research suggests epigenetic changes to the chromatin template begin at conception and continue as an iterative process enabling a progressive “memory” of prior developmental fate decisions. However, the biochemical nature of this memory is the subject of some debate. In the case of the H3K27 me3 mark, work by two independent laboratories has suggested that established H3K27 me3 attracts the *EED* component of the Polycomb Repressive Complex 2 (*PRC2*), and is required to stimulate the enzymatic activity necessary to maintain this mark on the daughter strands during incorporation of newly synthesized histones [79, 80]. In contrast, another study has suggested that only binding of the *Polycomb* complex through S-phase is required to propagate this post-translational modification [32]. We have previously observed depletion of H3K27 me3 at multiple loci in response to EtOH exposure; however, ChIP analysis of EZH2 binding failed to identify significant changes in *PRC2* localization [10]. The persisting reductions of this mark observed 10 days after an acute in vivo exposure, but not within in vitro neurosphere cultures maintained in the stem cell state, suggest recovery of this mark may be dependent upon cells being in a stem cell versus a differentiating state.

In our in vitro studies, we did not observe a consistent correlation between alcohol-induced alterations in histone post-translational modifications and changes in gene transcription. The large majority of alterations in chromatin structure we observed indicate an increase in marks associated with transcriptional repression at Day-7, yet our candidate genes demonstrated an increase in expression at this time. These in vitro observations add one more piece of data to suggest histone post-translational modifications, in isolation, are not likely causal in regulating transcription [31]. Therefore, the established lexicon of ‘activating’ and ‘repressive’ chromatin modifications is an over-simplification that should be curtailed. Basic principles of teratogenesis state that a teratogen must cause malformations through a specific mechanism during a period in which the conceptus is susceptible to said mechanism [81]. Embryonic stem cells have been derived with genetic deletions of the major enzymes controlling DNA methylation (*Dnmt1*^−/−^*Dnmt3a*^−/−^*Dnmt3b*^−/−^triple knockout), H3K27 me3 (Suz12^−/−^) and H3K9 me2/me3 [(*G9a*^−/−^*GLP*^−/−^ double knockout) (*Suv39h1*^−/−^/*Suv39h2*^−/−^double knockout) ESET/Setdb1 knockout^−/−^] [82–86]. In most cases, these cells continue to grow and demonstrate subtle changes in gene transcription. However, once induced to begin the process of differentiation, these cultures uniformly undergo apoptosis. Thus, perhaps the stem cell state is tolerant of major shifts in chromatin structure without associated perturbation of transcriptional control, while in contrast, differentiating cells become ‘locked in’ and are more reliant upon chromatin states to control gene expression. In animal models, correlation of acute EtOH exposures with major periods of organ growth indicate that different tissues are largely susceptible to alcohol-induced teratogenesis during specific developmental windows. It is thus tempting to speculate that differences in chromatin biology between pluripotent, differentiating and differentiated cells underlie some aspects of susceptibility to alcohol teratogenesis.

Our analysis of mouse cortices derived from EtOH-exposed, malformed pups clearly indicate alterations in chromatin structure are heritable and persist beyond the window of exposure. Importantly, these alcohol-induced changes in chromatin structure can be found within the regulatory regions of genes with clear links to the development of FASD clinical phenotypes (Fig. 7b, d) [36, 38]. Thus, our data strongly suggest acute alcohol exposures have the capacity to perturb the developmental program and contribute to EtOH-induced birth defects. As approximately 50 % of pregnancies in the United States are unplanned, many women inadvertently subject their children to acute prenatal EtOH exposures [87]. Therefore, a better understanding of the role of alcohol-induced epigenetic errors in the development program play in the etiology of FASDs will enhance our ability to develop clinical interventions and better diagnostics in the treatment of this condition.

## Methods

### Neural stem cell culture and EtOH exposure

All animal procedures were approved and conducted in accordance with the Institutional Animal Care and Use Committee at the Texas A&M College of Veterinary Medicine (protocol number 2014-0087), and the University of North Carolina. Derivation of primary mouse fetal cerebral cortical neuroepithelial stem cells have been described in detail previously [88]. Cells were cultured as free floating neurospheres in T75 flasks containing a 50/50 % mixture of Neurobasal media (Cat# 21103-049; Invitrogen) and DMEM F-12 (Cat# 11320-033; Invitrogen). This medium was supplemented with the N2-supplement (Cat# 17502-048; Invitrogen), B27 supplement (Cat# 17504-044; Invitrogen), 0.05 % TC grade BSA in PBS (Cat# A1933 Sigma), 2 mM l-glutamine (Cat# 25030-081; Invitrogen), 1× penicillin/streptomycin (Cat# 15140-122; Invitrogen), 20 µg/mL FGF basic (Cat# PMG0034; Invitrogen), 20 µg/mL EGF (Cat# PHG0311; Invitrogen), and 0.85 units/mL heparin (Cat# H3149; Sigma). Neurospheres were incubated at 37 °C, in a 5 % CO_2_ humidified environment. Medium was changed every 2 or 3 days depending on the level of confluence. Alcohol treatment groups were cultured in medium containing either 80, 120, 160, 240 mg/dL, or control cultures containing no EtOH. Cells were grown in flasks sealed with parafilm to prevent evaporation. Medium treatments were replaced every 48 h and samples collected for ChIP and RNA analysis at the indicated time points.

### Chromatin immunoprecipitation analysis

Cells were grown to 80 % confluence, washed twice in warm PBS, dissociated using Accutase (Cat# SCR005; Millipore), and resuspended in warm medium (DMEM F-12 Cat# 11320-033; Invitrogen) containing 0.1 volume crosslinking solution [89]. ChIP reactions were performed as described previously [35] followed by DNA purification using a Qiaquick PCR Cleanup kit (Cat# 28106; QIAGEN). Antibodies used include: anti-H3K4me3 (Cat# 04-745; Millipore), anti-H3K27me3 (Cat# 39155; Active Motif), anti-H3K9ac (Cat# 07-352; Millipore), and anti-H3K9me2 (Cat# 39239; Active Motif). Antibodies for modified histones were used at 1 µg/ChIP reaction. The concentration of IgG (Cat# SC-2027; Santa Cruz) was also used at 1 µg/ChIP. For analysis of candidate loci, real-time PCR was performed using the Dynamo Flash supermix (Cat# F-415XL; Thermo Scientific) according to the recommended protocol. Reactions were performed on a Bio-Rad CFX384 Touch PCR system. Primer sequences are listed in Additional file 3.

### Murine fetal forebrain chromatin immunoprecipitation analysis

IP injections of EtOH (2.9 g/kg) were administered to pregnant dams at GD7 as described previously [70]. Control dams were injected with a comparable volume of lactated Ringer’s solution. Embryos were harvested at GD17, and fetal mice scored for ocular and cortical patterning defects [70, 71]. After assessment, the left and right cortices were dissected and flash frozen. Cortices of a single brain were thawed and filtered into a single-cell suspension using gentle mechanical dissociation in a 100 μm cell strainer (Cat# 352360; Corning Life Sciences). Cells were washed twice with PBS containing protease inhibitor cocktail (Cat# 78437; Thermo Scientific) and resuspended in medium (DMEM F-12 Cat# 11320-033; Invitrogen) containing 0.1 volume crosslinking solution [89]. ChIP reactions were performed as described above.

### RNA analysis

Cultured cells were grown to 80 % confluence, washed twice in warm PBS, and dissociated with 1× trypsin (Accutase Cat# SCR005; Millipore). Cells were spun down, washed once in cold PBS, and RNA isolated using Trizol (Cat# 15596026; Invitrogen) according to the manufacturer’s protocol. One microgram of purified total RNA was treated with amplification grade DNase I (Cat# AMPD1; Sigma) according to the manufacturer’s recommendations, and 250 ng RNA seeded into a reverse transcription reaction using the SuperScriptII system (Cat# 18064-071; Invitrogen) by combining 1 µL random hexamer oligonucleotides (Cat# 48190011; Invitrogen), 1 µL 10 mM dNTP (Cat# 18427-013; Invitrogen), and 11 µL RNA plus water. This mixture was brought to 70 °C for 5 min and then cooled to room temperature. SuperScriptII reaction buffer, DTT, and SuperScriptII were then added according to manufacturer’s protocol, and the mixture brought to 25 °C for 5 min, 42 °C for 50 min, 45 °C for 20 min, 50 °C for 15 min, and then 70 °C for 5 min. Relative levels of candidate gene transcripts were analyzed using the Dynamo Flash mastermix according to the recommended protocol. Reactions were performed on a Bio-Rad CFX38. Primer sequences are listed in Additional file 3.

### Histone methyltransferase activity assay

Cells were dissociated, washed twice in PBS, pelleted, and nuclear extracts prepared. Briefly, cell pellets were initially resuspended in 200 µL of Buffer A (10 mM HEPES, 10 mM KCl, 0.1 mM EDTA). Following a 10-min incubation, samples were centrifuged at 20,000×*g* for 3 min at 4 °C. The supernatants were removed and nuclei resuspended in 30 µL Buffer B (20 mM HEPES, 0.4 M NaCl, 1 mM EDTA, 10 % glycerol). Samples were shaken at 1000 RPM for 2 h followed by centrifugation at 20,000×*g* for 5 min. H3K9 and H3K27 Methyltransferase activity were assayed using the EpiQuik HMT Activity Assay Kit (Cat# P-3005, P3003; Epigentek), according to the manufacturer’s instructions. Absorbance was measured on a Cary Eclipse microplate reader (Agilent Technologies) at a wavelength of 450 nm. Activity was calculated according to the manufacturer’s instructions.

### Analysis of cellular stress and apoptosis

#### Intracellular glutathione assay

One million cells were prepared in 1 mL warm DMEM F-12 (Cat# 11320-033; Invitrogen), and glutathione measured using an Intracellular GSH detection assay (Cat# ab112132; Abcam) following the manufacturer’s recommendations. Fluorescence was monitored using an Accuri C6 flow cytometer (BD Biosciences).

#### Lactate dehydrogenase assay

One million cells were collected and LDH levels quantified using a Lactate Dehydrogenase Activity Assay Kit (Cat# MAK066; Sigma), according to the recommended protocol. Measurements were taken using a Cary Eclipse microplate reader (Agilent Technologies) at a wavelength of 450 nm.

#### Annexin V apoptosis assay

Cells were examined using the Annexin V Apoptosis Detection Kit (APC; Cat# 88-8007; eBioscience). Five million cells were washed once in PBS and resuspended in binding buffer. 5 µL of Annexin V was added to 100 µL of cell suspension and incubated for 15 min at room temperature. Cells were then washed in PBS and resuspended in 200 µL of Binding Buffer. 5 µL of propidium iodide solution was added, and cells analyzed using an Accuri C6 flow cytometer (BD Biosciences).

#### TUNEL assay

Using a APO-BrdU TUNEL Assay Kit (Cat# A35127; Invitrogen), one million cells were fixed using paraformaldehyde and resuspended in 50 µL DNA labeling solution according to the manufacturer’s protocols. 500 µL of propidium iodide/RNase A staining buffer was added to each sample and incubated for an additional 30 min at room temperature in the dark. Samples were analyzed using an Accuri C6 flow cytometer (BD Biosciences).

### Analysis of DNA methylation and DNA hydroxymethylation

Genomic DNA was isolated from treated neurospheres using the DNeasy Blood and Tissue kit (Cat# 69506; QIAGEN) according to the recommended protocol. We utilized glucosylation of genomic DNA followed by methylation-sensitive qPCR (glucMS-qPCR) to quantify levels of 5-methyl-cytosine and 5-hydroxy-methyl-cytosine. Here, 30 µg of genomic DNA was treated with 30 units of T4 phage β-glucosyltransferase (T4 BGT, Cat# M0357S; NEB) at 37 °C overnight. Glycosylated genomic DNA was then digested with 100 units of MspI (Cat# R0106M; NEB) or 50 units of HpaII (Cat# R0171L; NEB), or no enzyme at 37 °C overnight. Reactions were inactivated by treatment with proteinase K (Cat# 19133; QIAGEN) at 40 °C for 30 min. The proteinase K was inactivated by incubation at 95 °C for 10 min. The HpaII- or MspI-resistant fractions were quantified by qPCR using primers designed around a single HpaII/MspI site. Primers listed in Additional file 3. Levels of 5-methyl-cytosine were determined by calculating differences in HpaII- vs. MspI-digested samples using the following formula: [% methylation = (2^(Uncut T4BGT treated − HpaII cut T4BGT treated)^ − 2^(Uncut T4BGT treated − MspI cut T4BGT treated)^) × 100]. Levels of 5-hydroxy-methyl-cytosine were determined by calculating the difference between glucosylated samples and genomic DNA digested with MspI using the following formula: [% hydroxymethylation = (2^(Uncut T4BGT treated − MspI cut T4BGT treated)^ − 2^(Uncut − MspI cut)^) × 100].

### Statistical analysis

For all experiments, statistical significance was set at alpha = 0.05.

For analysis of gene expression, the replicate cycle threshold (CT) values for each transcript were compiled and normalized to the geometric mean of the reference genes phosphoglycerate kinase 1 (*Pgk1*—NM_008828), glyceraldehyde 3-phosphate dehydrogenase (*Gapdh*—NM_008084), and hypoxanthine-phosphoribosyl transferase (*Hprt*—NM_013556). From our previous studies of 14 candidate reference genes in EtOH-exposed cultures, *Pgk1*, *Gapdh*, and *Hprt* have been validated as stable across the range of alcohol treatments utilized in this study [90]. Normalized expression levels were calculated using the DDCT method described previously [91]. Values from each biological replicate were transferred into the statistical analysis program GraphPad (GraphPad Software, Inc., La Jolla, CA, USA), verified for normality, and an analysis of variance (ANOVA) run to assay differences between experimental treatments. For samples with *p* values <0.05, we applied Tukey’s HSD analysis for multiple comparisons, and have marked statistically significant differences with an asterisk.

For quantitative analysis of candidate gene regulatory region enrichment in primary fetal cerebral cortical neuroepithelial stem cells, ChIP samples were first normalized to 1 % input, using the formula previously described by Mukhopadhyay et al. [92]. To independently examine alterations in each post-translational modification, the means from each independent sample were normalized to the control. The results of three independent experiments were then tabulated, cumulative means calculated and standard error of the mean derived. The statistical analysis package GraphPad was used to first measure the normality of samples using the D’Agostino–Pearson test. As several of the candidate genes did not exhibit a normal distribution, we quantified differences between control and EtOH-treated samples using the Wilcoxon signed rank test. This nonparametric test is applied when the population has unequal variances and cannot be assumed to be normally distributed. Importantly, this test has been widely employed in genome wide studies of histone variants, histone post-translational marks and transcription factor binding [93, 94].

For quantitative analysis of candidate gene regulatory region enrichment in fetal forebrains, ChIP samples were normalized to 1 % input, using the formula previously described [92]. The cumulative mean from each of the three independent experiments was calculated and the standard error of the mean derived. The statistical analysis package GraphPad was used to verify normality, and assay differences between each of the control, EtOH exposed—morphologically normal, and EtOH exposed—malformed samples using a two-way analysis of variance (ANOVA).

For quantitative analysis of DNA methylation, percentages of DNA 5-methylcytosine and 5-hydroxymethylcytosine derived from the formulas listed above were transferred into the statistical analysis package GraphPad, verified for normality, and an analysis of variance (ANOVA) run to assay differences between experimental treatments. A Student’s *t* test was run to assay differences between days 3 and 7 among all samples tested.

## Additional files

10.1186/s13072-015-0031-7 ChIP assay and quantitative PCR (qPCR) analysis of the individual candidate genes examined in Figure 1.
10.1186/s13072-015-0031-7 ChIP assay and quantitative PCR (qPCR) analysis of the individual candidate genes examined in Figure 7.
10.1186/s13072-015-0031-7 The primer sequences utilized in the ChIP assay and quantitative PCR (qPCR) and expression analysis.

## Abbreviations

EtOH: ethanol
FASDs: fetal alcohol spectrum disorders
FAS: fetal alcohol syndrome
ROS: reactive oxygen species
ChIP: chromatin immunoprecipitation
H3K4me3: trimethylated histone 3 lysine 4
H3K27me3: histone 3 lysine 27 trimethylation
H3K9ac: acetylated histone 3 lysine 9
H3K9me3: dimethylated histone 3 lysine 9
5mC: 5-methyl-cytosine
5hmC: 5-hydroxy-methyl-cytosine
Dnmt1: DNA methyltransferase 1
*GSH*: glutathione
LDH: lactate dehydrogenase
glucMS-qPCR: glucosylation of genomic DNA followed by methylation-sensitive qPCR
5′UTR: 5′ untranslated region
qRT-PCR: quantitative reverse transcriptase polymerase chain reaction
CNS: central nervous system
IP: intraperitoneal
ANOVA: analysis of variance
SEM: standard error of the mean

## References

[1] Feil R, Fraga MF (2011). Epigenetics and the environment: emerging patterns and implications. Nat Rev Genet.

[2] Mead EA, Sarkar DK (2014). Fetal alcohol spectrum disorders and their transmission through genetic and epigenetic mechanisms. Front Genet.

[3] Resendiz M, Mason S, Lo CL, Zhou FC (2014). Epigenetic regulation of the neural transcriptome and alcohol interference during development. Front Genet.

[4] Ungerer M, Knezovich J, Ramsay M (2013). In utero alcohol exposure, epigenetic changes, and their consequences. Alcohol Res.

[5] Garro AJ, McBeth DL, Lima V, Lieber CS (1991). Ethanol consumption inhibits fetal DNA methylation in mice: implications for the fetal alcohol syndrome. Alcohol Clin Exp Res.

[6] Bielawski DM, Zaher FM, Svinarich DM, Abel EL (2002). Paternal alcohol exposure affects sperm cytosine methyltransferase messenger RNA levels. Alcohol Clin Exp Res.

[7] Kaminen-Ahola N, Ahola A, Maga M, Mallitt KA, Fahey P, Cox TC (2010). Maternal ethanol consumption alters the epigenotype and the phenotype of offspring in a mouse model. PLoS Genet.

[8] Zhou FC, Chen Y, Love A (2011). Cellular DNA methylation program during neurulation and its alteration by alcohol exposure. Birth Defects Res A Clin Mol Teratol.

[9] Knezovich JG, Ramsay M (2012). The effect of preconception paternal alcohol exposure on epigenetic remodeling of the h19 and rasgrf1 imprinting control regions in mouse offspring. Front Genet.

[10] Veazey KJ, Carnahan MN, Muller D, Miranda RC, Golding MC (2013). Alcohol-induced epigenetic alterations to developmentally crucial genes regulating neural stemness and differentiation. Alcohol Clin Exp Res.

[11] Pal-Bhadra M, Bhadra U, Jackson DE, Mamatha L, Park PH, Shukla SD (2007). Distinct methylation patterns in histone H3 at lys-4 and lys-9 correlate with up- and down-regulation of genes by ethanol in hepatocytes. Life Sci.

[12] Bekdash RA, Zhang C, Sarkar DK (2013). Gestational choline supplementation normalized fetal alcohol-induced alterations in histone modifications, DNA methylation, and proopiomelanocortin (POMC) gene expression in β-endorphin-producing POMC neurons of the hypothalamus. Alcohol Clin Exp Res.

[13] Moonat S, Sakharkar AJ, Zhang H, Tang L, Pandey SC (2013). Aberrant histone deacetylase2-mediated histone modifications and synaptic plasticity in the amygdala predisposes to anxiety and alcoholism. Biol Psychiatry.

[14] Pan B, Zhu J, Lv T, Sun H, Huang X, Tian J (2014). Alcohol consumption during gestation causes histone3 lysine9 hyperacetylation and an alternation of expression of heart development-related genes in mice. Alcohol Clin Exp Res.

[15] Brocardo PS, Gil-Mohapel J, Christie BR (2011). The role of oxidative stress in fetal alcohol spectrum disorders. Brain Res Rev.

[16] Pikkarainen PH (1971). Metabolism of ethanol and acetaldehyde in perfused human fetal liver. Life Sci II.

[17] Waltman R, Iniquez ES (1972). Placental transfer of ethanol and its elimination at term. Obstet Gynecol.

[18] Brien JF, Loomis CW, Tranmer J, McGrath M (1983). Disposition of ethanol in human maternal venous blood and amniotic fluid. Am J Obstet Gynecol.

[19] Brzezinski MR, Boutelet-Bochan H, Person RE, Fantel AG, Juchau MR (1999). Catalytic activity and quantitation of cytochrome P-450 2E1 in prenatal human brain. J Pharmacol Exp Ther.

[20] Zakhari S (2013). Alcohol metabolism and epigenetics changes. Alcohol Res.

[21] Chia N, Wang L, Lu X, Senut MC, Brenner C, Ruden DM (2011). Hypothesis: environmental regulation of 5-hydroxymethylcytosine by oxidative stress. Epigenetics.

[22] Garro AJ, Espina N, McBeth D, Wang SL, Wu-Wang CY (1992). Effects of alcohol consumption on DNA methylation reactions and gene expression: implications for increased cancer risk. Eur J Cancer Prev.

[23] Halsted CH, Villanueva JA, Devlin AM, Niemelä O, Parkkila S, Garrow TA (2002). Folate deficiency disturbs hepatic methionine metabolism and promotes liver injury in the ethanol-fed micropig. Proc Natl Acad Sci USA.

[24] Haycock PC, Ramsay M (2009). Exposure of mouse embryos to ethanol during preimplantation development: effect on DNA methylation in the h19 imprinting control region. Biol Reprod.

[25] Ouko LA, Shantikumar K, Knezovich J, Haycock P, Schnugh DJ, Ramsay M (2009). Effect of alcohol consumption on cpg methylation in the differentially methylated regions of H19 and IG-DMR in male gametes: implications for fetal alcohol spectrum disorders. Alcohol Clin Exp Res.

[26] Liu Y, Balaraman Y, Wang G, Nephew KP, Zhou FC (2009). Alcohol exposure alters DNA methylation profiles in mouse embryos at early neurulation. Epigenetics.

[27] Zhou FC, Balaraman Y, Teng M, Liu Y, Singh RP, Nephew KP (2011). Alcohol alters DNA methylation patterns and inhibits neural stem cell differentiation. Alcohol Clin Exp Res.

[28] Downing C, Johnson TE, Larson C, Leakey TI, Siegfried RN, Rafferty TM, Cooney CA (2011). Subtle decreases in DNA methylation and gene expression at the mouse igf2 locus following prenatal alcohol exposure: effects of a methyl-supplemented diet. Alcohol.

[29] Kim JS, Shukla SD (2005). Histone h3 modifications in rat hepatic stellate cells by ethanol. Alcohol Alcohol.

[30] Park PH, Lim RW, Shukla SD (2005). Involvement of histone acetyltransferase (HAT) in ethanol-induced acetylation of histone H3 in hepatocytes: potential mechanism for gene expression. Am J Physiol Gastrointest Liver Physiol.

[31] Henikoff S, Shilatifard A (2011). Histone modification: cause or cog?. Trends Genet.

[32] Petruk S, Sedkov Y, Johnston DM, Hodgson JW, Black KL, Kovermann SK (2012). TrxG and pcg proteins but not methylated histones remain associated with DNA through replication. Cell.

[33] Nestler EJ (2014). Epigenetic mechanisms of drug addiction. Neuropharmacology.

[34] White AM, Kraus CL, Swartzwelder H (2006). Many college freshmen drink at levels far beyond the binge threshold. Alcohol Clin Exp Res.

[35] Golding MC, Zhang L, Mann MR (2010). Multiple epigenetic modifiers induce aggressive viral extinction in extraembryonic endoderm stem cells. Cell Stem Cell.

[36] Rifas L, Towler DA, Avioli LV (1997). Gestational exposure to ethanol suppresses msx2 expression in developing mouse embryos. Proc Natl Acad Sci USA.

[37] Wentzel P, Eriksson UJ (2009). Altered gene expression in neural crest cells exposed to ethanol in vitro. Brain Res.

[38] Godin EA, Dehart DB, Parnell SE, O’Leary-Moore SK, Sulik KK (2011). Ventromedian forebrain dysgenesis follows early prenatal ethanol exposure in mice. Neurotoxicol Teratol.

[39] Zhou FC, Zhao Q, Liu Y, Goodlett CR, Liang T, McClintick JN (2011). Alteration of gene expression by alcohol exposure at early neurulation. BMC Genomics.

[40] Mozzetta C, Pontis J, Fritsch L, Robin P, Portoso M, Proux C (2014). The histone H3 lysine 9 methyltransferases g9a and GLP regulate polycomb repressive complex 2-mediated gene silencing. Mol Cell.

[41] Landeira D, Sauer S, Poot R, Dvorkina M, Mazzarella L, Jørgensen HF (2010). Jarid2 is a PRC2 component in embryonic stem cells required for multi-lineage differentiation and recruitment of PRC1 and RNA polymerase II to developmental regulators. Nat Cell Biol.

[42] Li G, Margueron R, Ku M, Chambon P, Bernstein BE, Reinberg D (2010). Jarid2 and PRC2, partners in regulating gene expression. Genes Dev.

[43] Mejetta S, Morey L, Pascual G, Kuebler B, Mysliwiec MR, Lee Y (2011). Jarid2 regulates mouse epidermal stem cell activation and differentiation. EMBO J.

[44] Dong J, Sulik KK, Chen SY (2008). Nrf2-mediated transcriptional induction of antioxidant response in mouse embryos exposed to ethanol in vivo: implications for the prevention of fetal alcohol spectrum disorders. Antioxid Redox Signal.

[45] Bosch-Presegué L, Raurell-Vila H, Marazuela-Duque A, Kane-Goldsmith N, Valle A, Oliver J (2011). Stabilization of suv39h1 by sirt1 is part of oxidative stress response and ensures genome protection. Mol Cell.

[46] Li E, Bestor TH, Jaenisch R (1992). Targeted mutation of the DNA methyltransferase gene results in embryonic lethality. Cell.

[47] Okano M, Bell DW, Haber DA, Li E (1999). DNA methyltransferases dnmt3a and dnmt3b are essential for de novo methylation and mammalian development. Cell.

[48] Cao R, Wang L, Wang H, Xia L, Erdjument-Bromage H, Tempst P (2002). Role of histone H3 lysine 27 methylation in polycomb-group silencing. Science.

[49] Schultz DC, Ayyanathan K, Negorev D, Maul GG, Rauscher FJ (2002). SETDB1: a novel kap-1-associated histone H3, lysine 9-specific methyltransferase that contributes to hp1-mediated silencing of euchromatic genes by KRAB zinc-finger proteins. Genes Dev.

[50] Tachibana M, Sugimoto K, Nozaki M, Ueda J, Ohta T, Ohki M (2002). G9a histone methyltransferase plays a dominant role in euchromatic histone H3 lysine 9 methylation and is essential for early embryogenesis. Genes Dev.

[51] Whetstine JR, Nottke A, Lan F, Huarte M, Smolikov S, Chen Z (2006). Reversal of histone lysine trimethylation by the JMJD2 family of histone demethylases. Cell.

[52] Yamane K, Toumazou C, Tsukada Y, Erdjument-Bromage H, Tempst P, Wong J, Zhang Y (2006). JHDM2A, a jmjc-containing H3K9 demethylase, facilitates transcription activation by androgen receptor. Cell.

[53] Bostick M, Kim JK, Estève PO, Clark A, Pradhan S, Jacobsen SE (2007). UHRF1 plays a role in maintaining DNA methylation in mammalian cells. Science.

[54] Wissmann M, Yin N, Müller JM, Greschik H, Fodor BD, Jenuwein T (2007). Cooperative demethylation by JMJD2C and LSD1 promotes androgen receptor-dependent gene expression. Nat Cell Biol.

[55] Laurent B, Ruitu L, Murn J, Hempel K, Ferrao R, Xiang Y (2015). A specific LSD1/KDM1A isoform regulates neuronal differentiation through H3K9 demethylation. Mol Cell.

[56] Tahiliani M, Koh KP, Shen Y, Pastor WA, Bandukwala H, Brudno Y (2009). Conversion of 5-methylcytosine to 5-hydroxymethylcytosine in mammalian DNA by MLL partner TET1. Science.

[57] Kriaucionis S, Heintz N (2009). The nuclear DNA base 5-hydroxymethylcytosine is present in purkinje neurons and the brain. Science.

[58] Viré E, Brenner C, Deplus R, Blanchon L, Fraga M, Didelot C (2006). The polycomb group protein EZH2 directly controls DNA methylation. Nature.

[59] Ciccone DN, Su H, Hevi S, Gay F, Lei H, Bajko J (2009). KDM1B is a histone H3K4 demethylase required to establish maternal genomic imprints. Nature.

[60] Rothbart SB, Krajewski K, Nady N, Tempel W, Xue S, Badeaux AI (2012). Association of UHRF1 with methylated H3K9 directs the maintenance of DNA methylation. Nat Struct Mol Biol.

[61] Sakharkar AJ, Tang L, Zhang H, Chen Y, Grayson DR, Pandey SC (2014). Effects of acute ethanol exposure on anxiety measures and epigenetic modifiers in the extended amygdala of adolescent rats. Int J Neuropsychopharmacol.

[62] Tan L, Xiong L, Xu W, Wu F, Huang N, Xu Y (2013). Genome-wide comparison of DNA hydroxymethylation in mouse embryonic stem cells and neural progenitor cells by a new comparative hmedip-seq method. Nucleic Acids Res.

[63] Okashita N, Kumaki Y, Ebi K, Nishi M, Okamoto Y, Nakayama M (2014). PRDM14 promotes active DNA demethylation through the ten-eleven translocation (TET)-mediated base excision repair pathway in embryonic stem cells. Development.

[64] Irwin RE, Thakur A, O’Neill KM, Walsh CP (2014). 5-Hydroxymethylation marks a class of neuronal gene regulated by intragenic methylcytosine levels. Genomics.

[65] Mann MR, Lee SS, Doherty AS, Verona RI, Nolen LD, Schultz RM, Bartolomei MS (2004). Selective loss of imprinting in the placenta following preimplantation development in culture. Development.

[66] Liang G, Lin JC, Wei V, Yoo C, Cheng JC, Nguyen CT (2004). Distinct localization of histone H3 acetylation and H3-K4 methylation to the transcription start sites in the human genome. Proc Natl Acad Sci USA.

[67] Zhou Z, Yuan Q, Mash DC, Goldman D (2011). Substance-specific and shared transcription and epigenetic changes in the human hippocampus chronically exposed to cocaine and alcohol. Proc Natl Acad Sci USA.

[68] Ponomarev I, Wang S, Zhang L, Harris RA, Mayfield RD (2012). Gene coexpression networks in human brain identify epigenetic modifications in alcohol dependence. J Neurosci.

[69] Sulik KK, Johnston MC, Webb MA (1981). Fetal alcohol syndrome: embryogenesis in a mouse model. Science.

[70] Godin EA, O’Leary-Moore SK, Khan AA, Parnell SE, Ament JJ, Dehart DB (2010). Magnetic resonance microscopy defines ethanol-induced brain abnormalities in prenatal mice: effects of acute insult on gestational day 7. Alcohol Clin Exp Res.

[71] Parnell SE, Dehart DB, Wills TA, Chen SY, Hodge CW, Besheer J (2006). Maternal oral intake mouse model for fetal alcohol spectrum disorders: ocular defects as a measure of effect. Alcohol Clin Exp Res.

[72] Fox DJ, Pettygrove S, Cunniff C, O’Leary LA, Gilboa SM, Bertrand J (2015). Fetal alcohol syndrome among children aged 7–9 years—Arizona, Colorado, and New York, 2010. MMWR Morb Mortal Wkly Rep.

[73] Qiang M, Denny A, Lieu M, Carreon S, Li J (2011). Histone H3K9 modifications are a local chromatin event involved in ethanol-induced neuroadaptation of the NR2B gene. Epigenetics.

[74] Subbanna S, Nagre NN, Shivakumar M, Umapathy NS, Psychoyos D, Basavarajappa BS (2014). Ethanol induced acetylation of histone at g9a exon1 and g9a-mediated histone H3 dimethylation leads to neurodegeneration in neonatal mice. Neuroscience.

[75] Ragunathan K, Jih G, Moazed D (2015). Epigenetics. Epigenetic inheritance uncoupled from sequence-specific recruitment. Science.

[76] Audergon PN, Catania S, Kagansky A, Tong P, Shukla M, Pidoux AL, Allshire RC (2015). Epigenetics. Restricted epigenetic inheritance of H3K9 methylation. Science.

[77] Clarke-Harris R, Wilkin TJ, Hosking J, Pinkney J, Jeffery AN, Metcalf BS (2014). PGC1α promoter methylation in blood at 5–7 years predicts adiposity from 9 to 14 years (earlybird 50). Diabetes.

[78] Zeisel SH (2011). What choline metabolism can tell us about the underlying mechanisms of fetal alcohol spectrum disorders. Mol Neurobiol.

[79] Hansen KH, Bracken AP, Pasini D, Dietrich N, Gehani SS, Monrad A (2008). A model for transmission of the h3k27me3 epigenetic mark. Nat Cell Biol.

[80] Margueron R, Justin N, Ohno K, Sharpe ML, Son J, Drury WJ (2009). Role of the polycomb protein EED in the propagation of repressive histone marks. Nature.

[81] Karnofsky DA (1965). Drugs, as teratogens, in animals and man. Annu Rev Pharmacol.

[82] Peters AH, Kubicek S, Mechtler K, O’Sullivan RJ, Derijck AA, Perez-Burgos L (2003). Partitioning and plasticity of repressive histone methylation states in mammalian chromatin. Mol Cell.

[83] Tachibana M, Ueda J, Fukuda M, Takeda N, Ohta T, Iwanari H (2005). Histone methyltransferases g9a and GLP form heteromeric complexes and are both crucial for methylation of euchromatin at H3-K9. Genes Dev.

[84] Tsumura A, Hayakawa T, Kumaki Y, Takebayashi S, Sakaue M, Matsuoka C (2006). Maintenance of self-renewal ability of mouse embryonic stem cells in the absence of DNA methyltransferases dnmt1, dnmt3a and dnmt3b. Genes Cells.

[85] Pasini D, Bracken AP, Hansen JB, Capillo M, Helin K (2007). The polycomb group protein suz12 is required for embryonic stem cell differentiation. Mol Cell Biol.

[86] Matsui T, Leung D, Miyashita H, Maksakova IA, Miyachi H, Kimura H (2010). Proviral silencing in embryonic stem cells requires the histone methyltransferase ESET. Nature.

[87] Henshaw SK (1998). Unintended pregnancy in the united states. Fam Plann Perspect.

[88] Miranda RC, Santillano DR, Camarillo C, Dohrman D (2008). Modeling the impact of alcohol on cortical development in a dish: strategies from mapping neural stem cell fate. Methods Mol Biol.

[89] Kondo Y, Shen L, Yan PS, Huang TH, Issa JP (2004). Chromatin immunoprecipitation microarrays for identification of genes silenced by histone H3 lysine 9 methylation. Proc Natl Acad Sci USA.

[90] Carnahan MN, Veazey KJ, Muller D, Tingling JD, Miranda RC, Golding MC (2013). Identification of cell-specific patterns of reference gene stability in quantitative reverse-transcriptase polymerase chain reaction studies of embryonic, placental and neural stem models of prenatal ethanol exposure. Alcohol.

[91] Schmittgen TD, Livak KJ (2008). Analyzing real-time PCR data by the comparative C(T) method. Nat Protoc.

[92] Mukhopadhyay A, Deplancke B, Walhout AJ, Tissenbaum HA (2008). Chromatin immunoprecipitation (chip) coupled to detection by quantitative real-time PCR to study transcription factor binding to DNA in caenorhabditis elegans. Nat Protoc.

[93] Liu B, Yi J, Sv A, Lan X, Ma Y, Huang TH (2013). QChIPat: a quantitative method to identify distinct binding patterns for two biological chip-seq samples in different experimental conditions. BMC Genom.

[94] Nakato R, Itoh T, Shirahige K (2013). DROMPA: easy-to-handle peak calling and visualization software for the computational analysis and validation of chip-seq data. Genes Cells.

