# Supplementary material for: Dose-dependent alcohol-induced alterations in chromatin structure persist beyond the window of exposure and correlate with fetal alcohol syndrome birth defects
Source: Epigenetics Chromatin. 2015 Sep 28;8:39. doi: 10.1186/s13072-015-0031-7 (PMC4587584; doi:10.1186/s13072-015-0031-7)
Supplement: Supplementary file 1 — 10.1186/s13072-015-0031-7 ChIP assay and quantitative PCR (qPCR) analysis of the individual candidate genes examined in Figure 1. [file 13072_2015_31_MOESM1_ESM.pdf]

Additional Figure 1

Alcohol-induced epigenetic alterations in H3K4 me3, H3K9 ac, H3K9 me2, and H3K27 me3 in primary fetal cerebral cortical neuroepithelial stem cells. Stem cells were cultured in the presence of 160 mg/dL or 240 mg/dL EtOH for 3 days, followed by a 4 day recovery in medium lacking EtOH. Samples were collected at days 3 and 7, and examined for changes in the indicated post-translational histone modifications using chromatin immunoprecipitation followed by quantitative PCR (ChIP-qPCR). Primers were designed to fall within 250 base pairs of the transcriptional start site. Results are graphed relative to the control. Within the 3 separate biological replicates (N=3), 3 ChIPs were performed, and 2 qPCR replicates performed on each independent ChIP. Statistical measures were conducted using the Wilcoxon Signed Rank nonparametric test. *p<0.05; **p<0.01; ***p<0.001; ****p<0.0001.

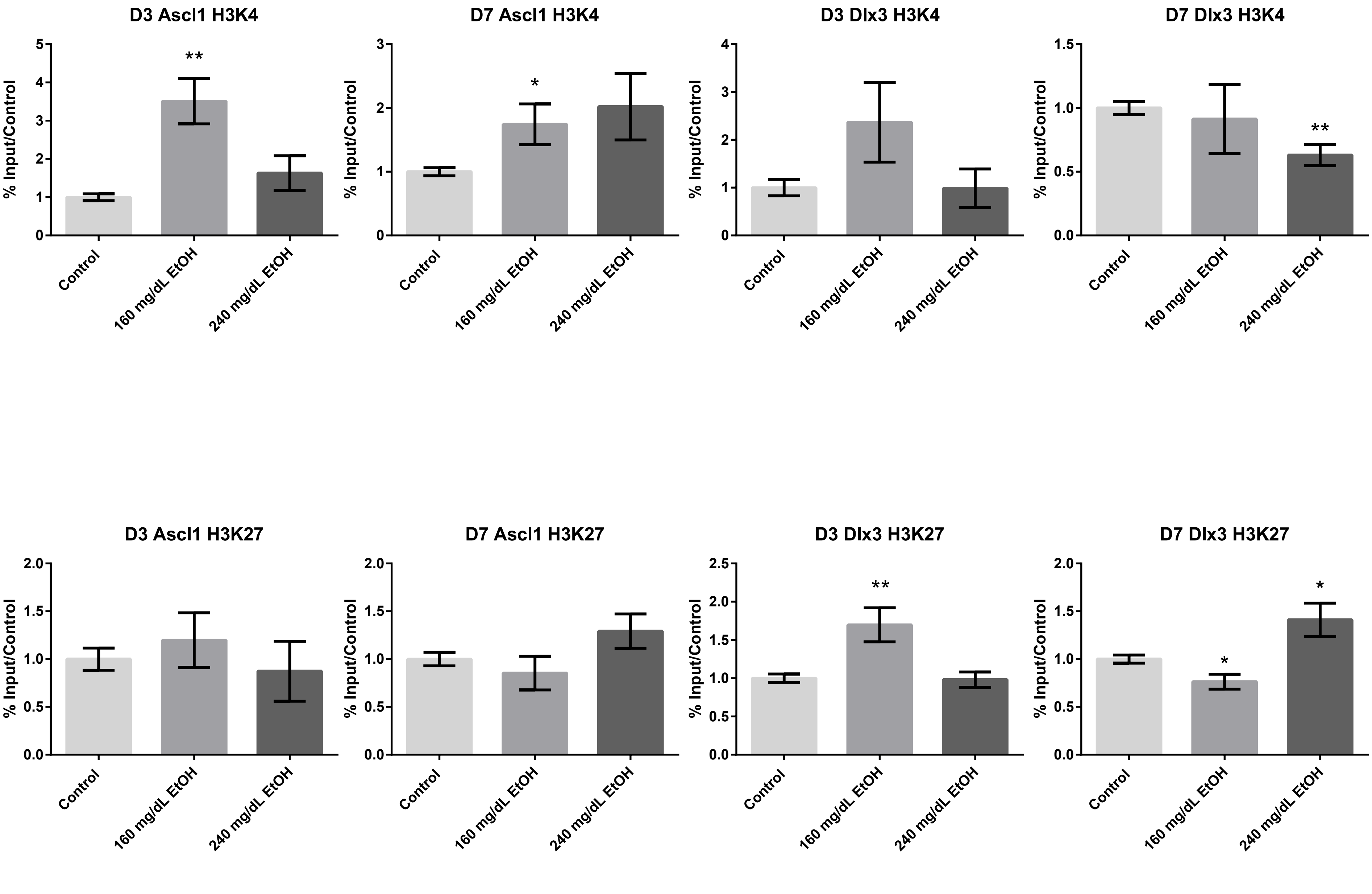
D3 Ascl1 H3K4
D7 Ascl1 H3K4
D3 Dlx3 H3K4
D7 Dlx3 H3K4
D3 Ascl1 H3K27
D7 Ascl1 H3K27
D3 Dlx3 H3K27
D7 Dlx3 H3K27
% Input/Control
Control
160 mg/dL EtOH
240 mg/dL EtOH
**
*
0
1
2
3
4
5
0.0
0.5
1.0
1.5
2.0
2.5

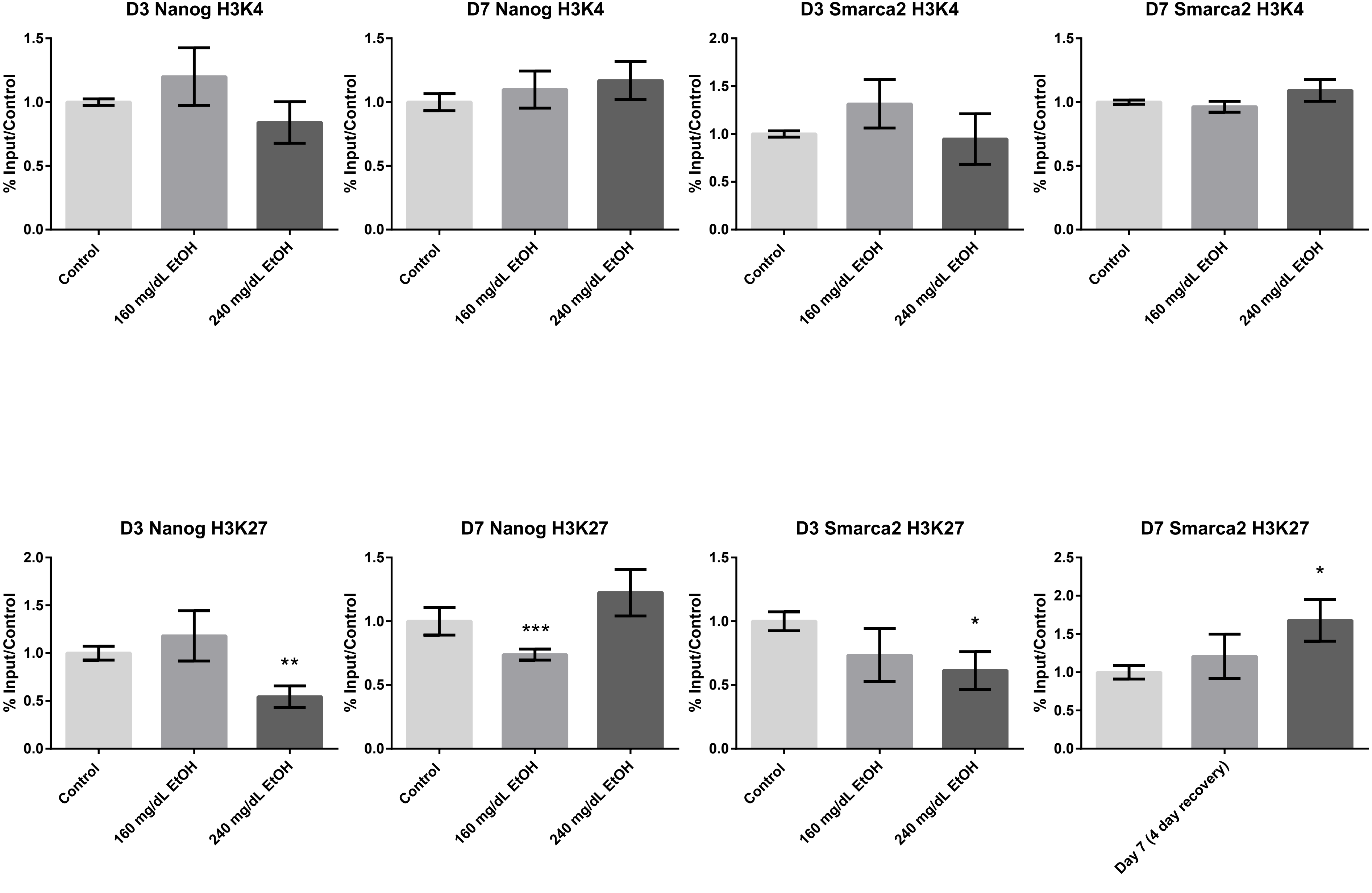
D3 Nanog H3K4
D7 Nanog H3K4
D3 Smarca2 H3K4
D7 Smarca2 H3K4
D3 Nanog H3K27
D7 Nanog H3K27
D3 Smarca2 H3K27
D7 Smarca2 H3K27
% Input/Control
Control
160 mg/dL EtOH
240 mg/dL EtOH
Day 7 (4 day recovery)
**
***
*
*

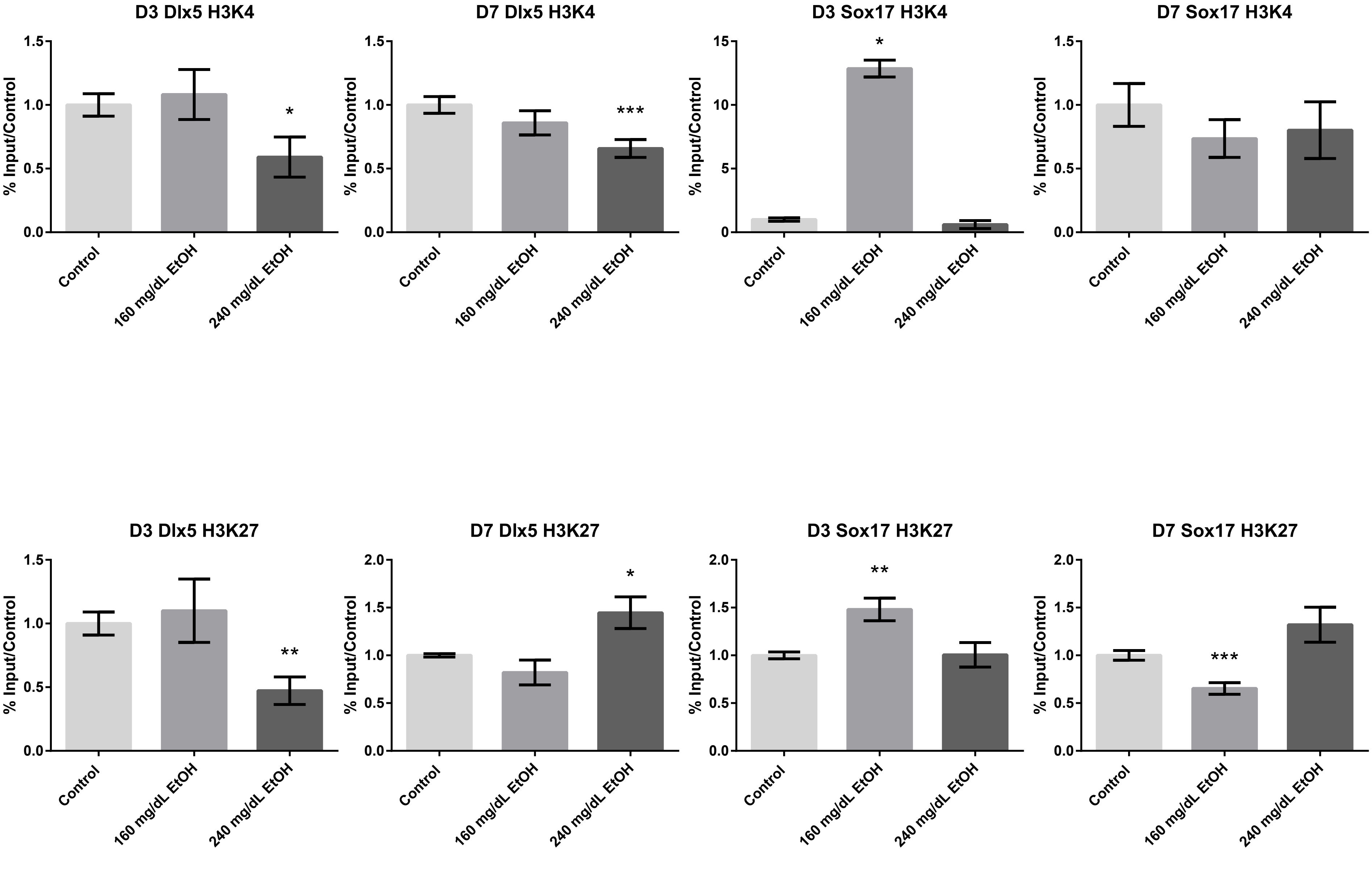
D3 Dlx5 H3K4
D7 Dlx5 H3K4
D3 Sox17 H3K4
D7 Sox17 H3K4
D3 Dlx5 H3K27
D7 Dlx5 H3K27
D3 Sox17 H3K27
D7 Sox17 H3K27
% Input/Control
Control
160 mg/dL EtOH
240 mg/dL EtOH

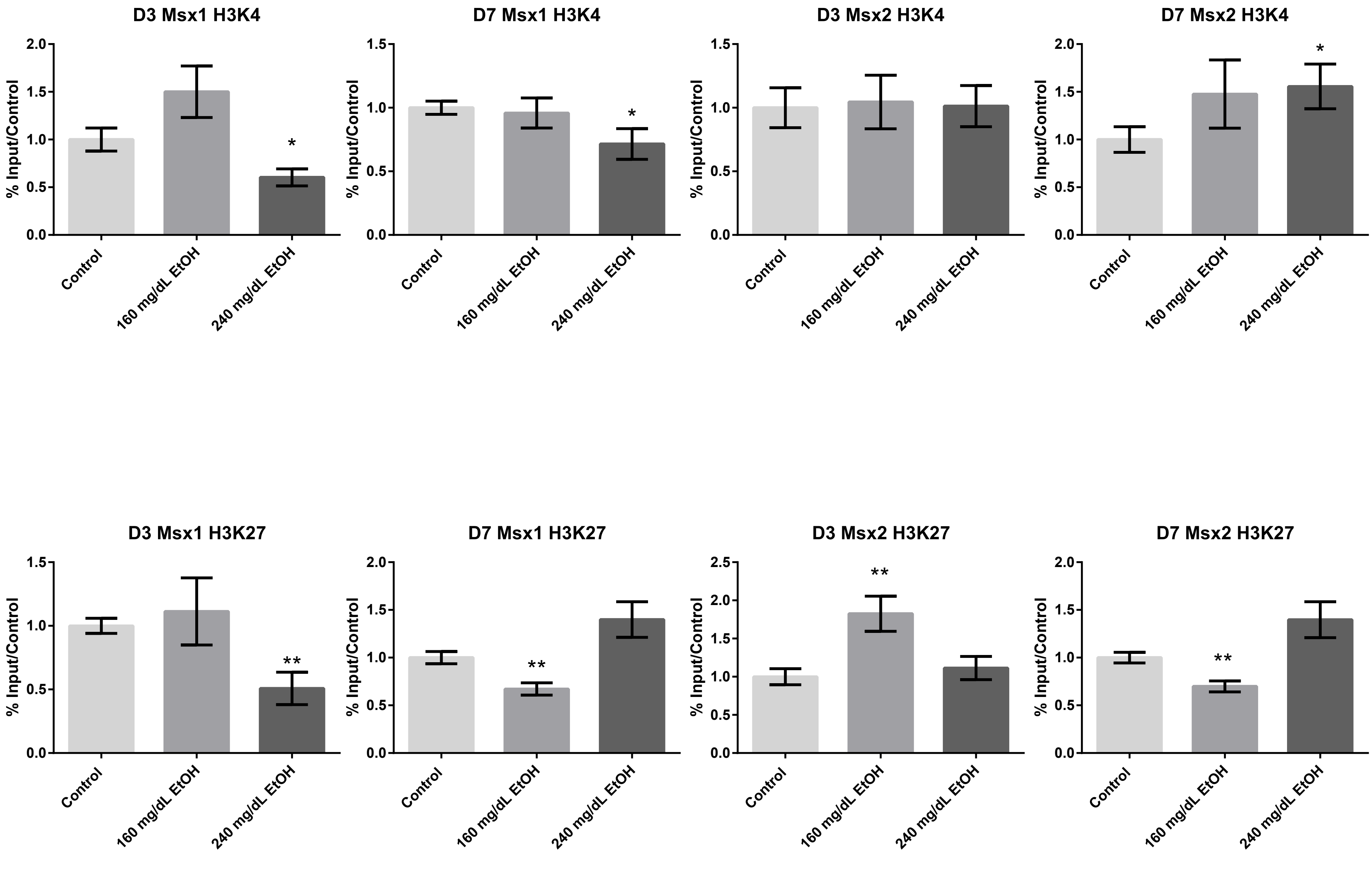

D3 Msx1 H3K4
D7 Msx1 H3K4
D3 Msx2 H3K4
D7 Msx2 H3K4
D3 Msx1 H3K27
D7 Msx1 H3K27
D3 Msx2 H3K27
D7 Msx2 H3K27
% Input/Control
Control
160 mg/dL EtOH
240 mg/dL EtOH
*
**
0.0
0.5
1.0
1.5
2.0
2.5

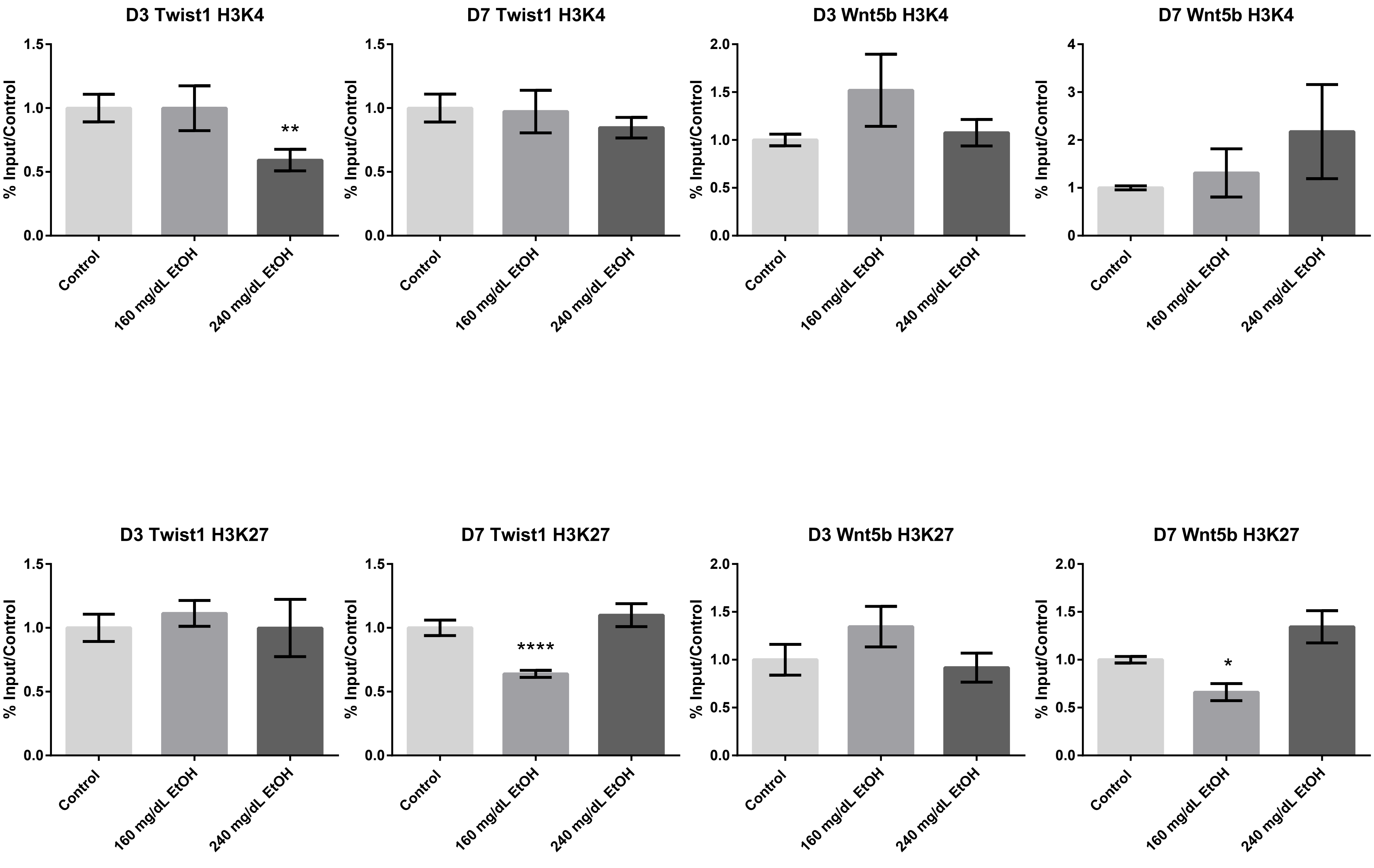
D3 Twist1 H3K4
D7 Twist1 H3K4
D3 Wnt5b H3K4
D7 Wnt5b H3K4
D3 Twist1 H3K27
D7 Twist1 H3K27
D3 Wnt5b H3K27
D7 Wnt5b H3K27
% Input/Control
Control
160 mg/dL EtOH
240 mg/dL EtOH
**
****
*

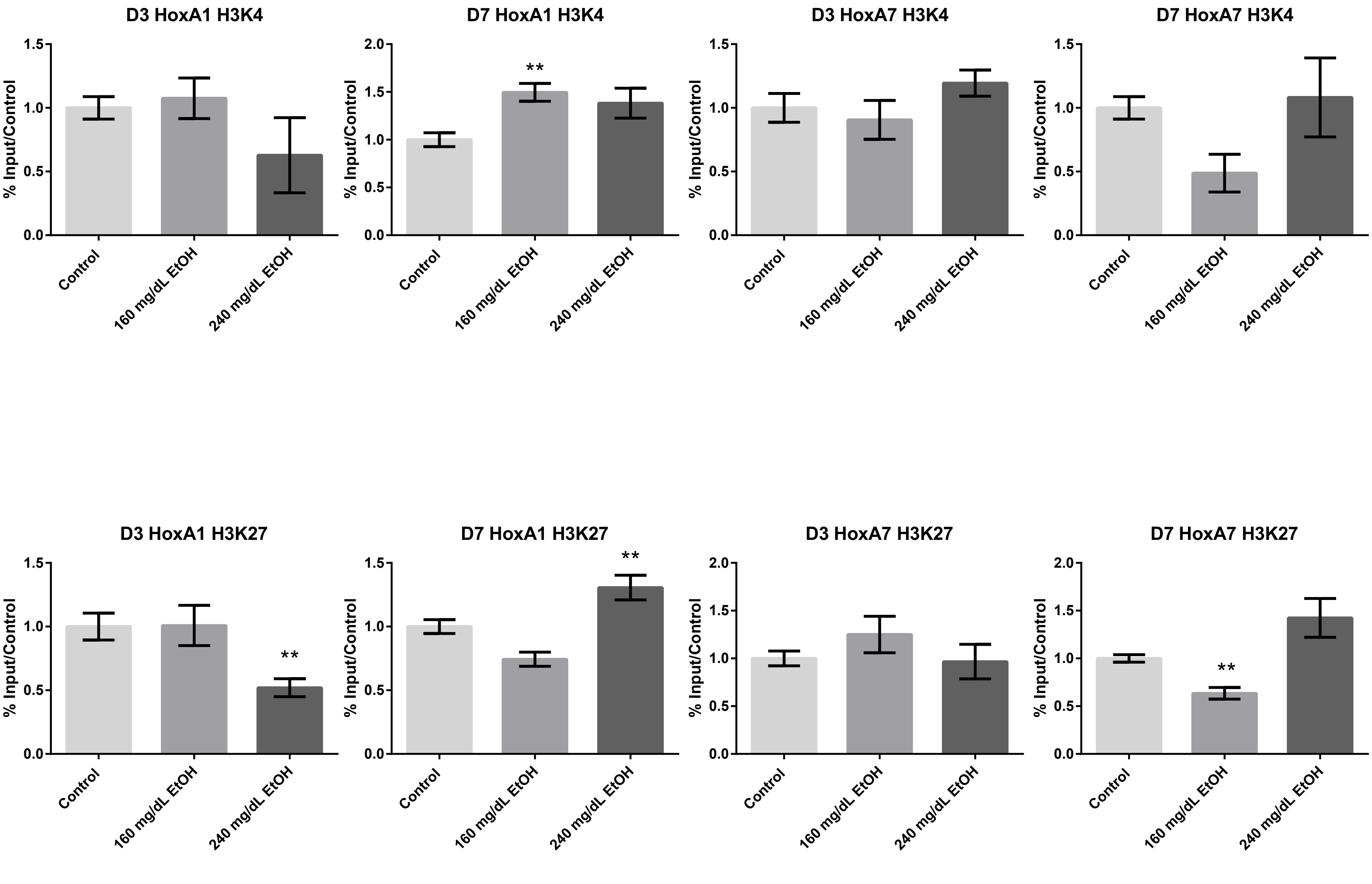
D3 HoxA1 H3K4
D7 HoxA1 H3K4
D3 HoxA7 H3K4
D7 HoxA7 H3K4
D3 HoxA1 H3K27
D7 HoxA1 H3K27
D3 HoxA7 H3K27
D7 HoxA7 H3K27
% Input/Control
Control
160 mg/dL EtOH
240 mg/dL EtOH
**

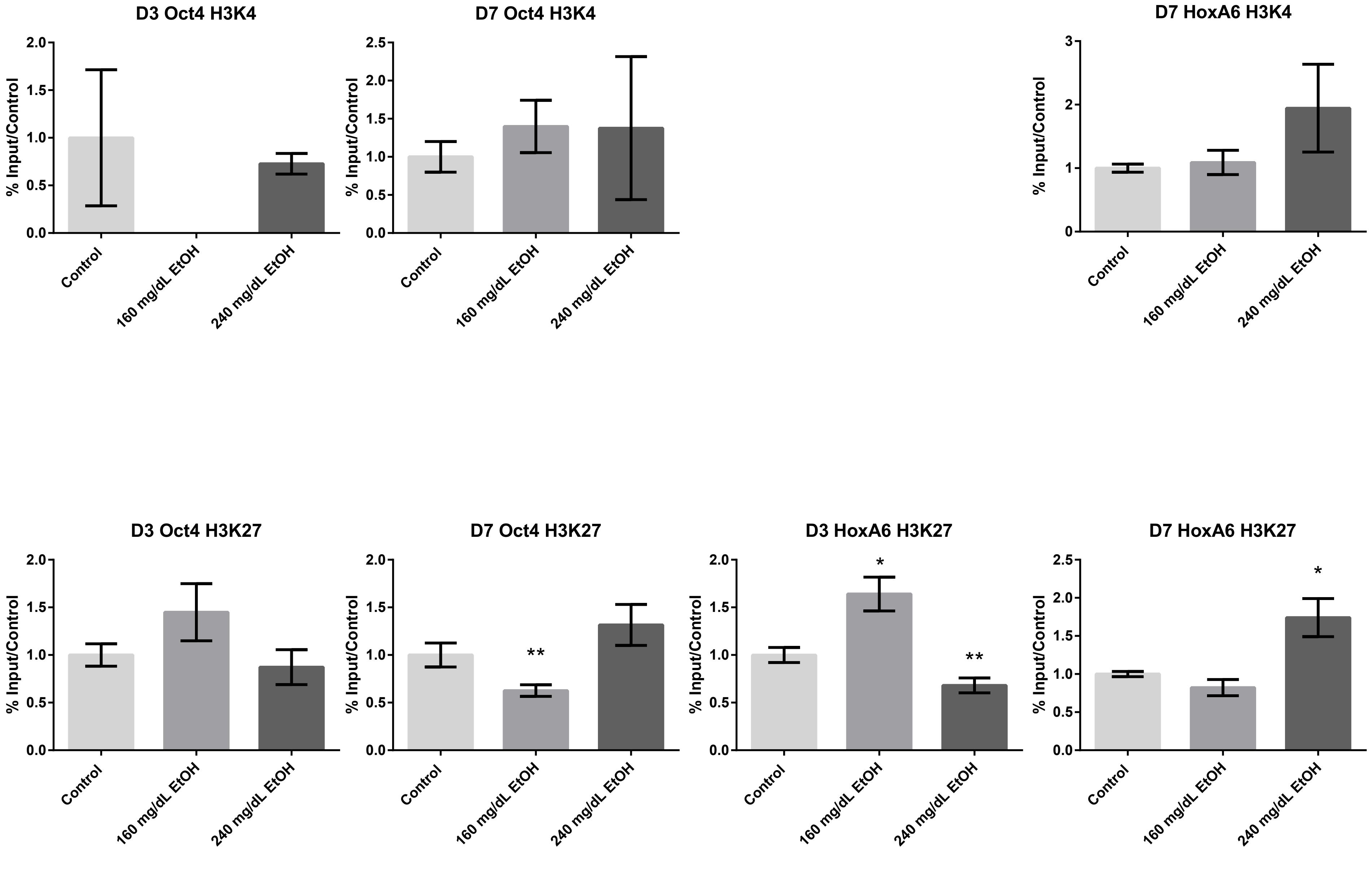

D3 Oct4 H3K4
D7 Oct4 H3K4
D7 HoxA6 H3K4
D3 Oct4 H3K27
D7 Oct4 H3K27
D3 HoxA6 H3K27
D7 HoxA6 H3K27
% Input/Control
Control
160 mg/dL EtOH
240 mg/dL EtOH
0.0
0.5
1.0
1.5
2.0
2.5
0
1
2
3
*
**

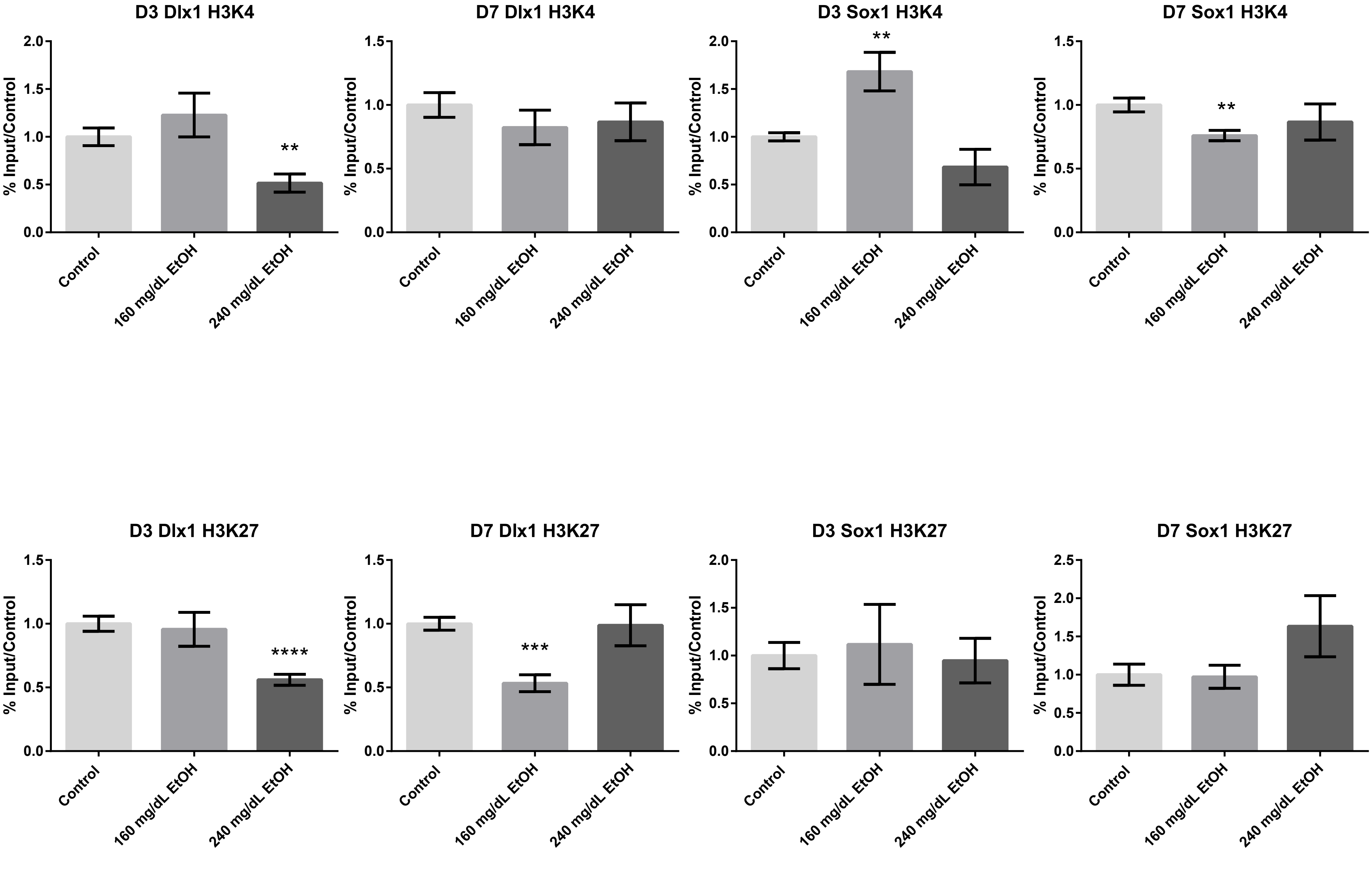
D3 Dlx1 H3K4
D7 Dlx1 H3K4
D3 Sox1 H3K4
D7 Sox1 H3K4
D3 Dlx1 H3K27
D7 Dlx1 H3K27
D3 Sox1 H3K27
D7 Sox1 H3K27
% Input/Control
0.0
0.5
1.0
1.5
2.0
2.5
Control
160 mg/dL EtOH
240 mg/dL EtOH
**
***
****

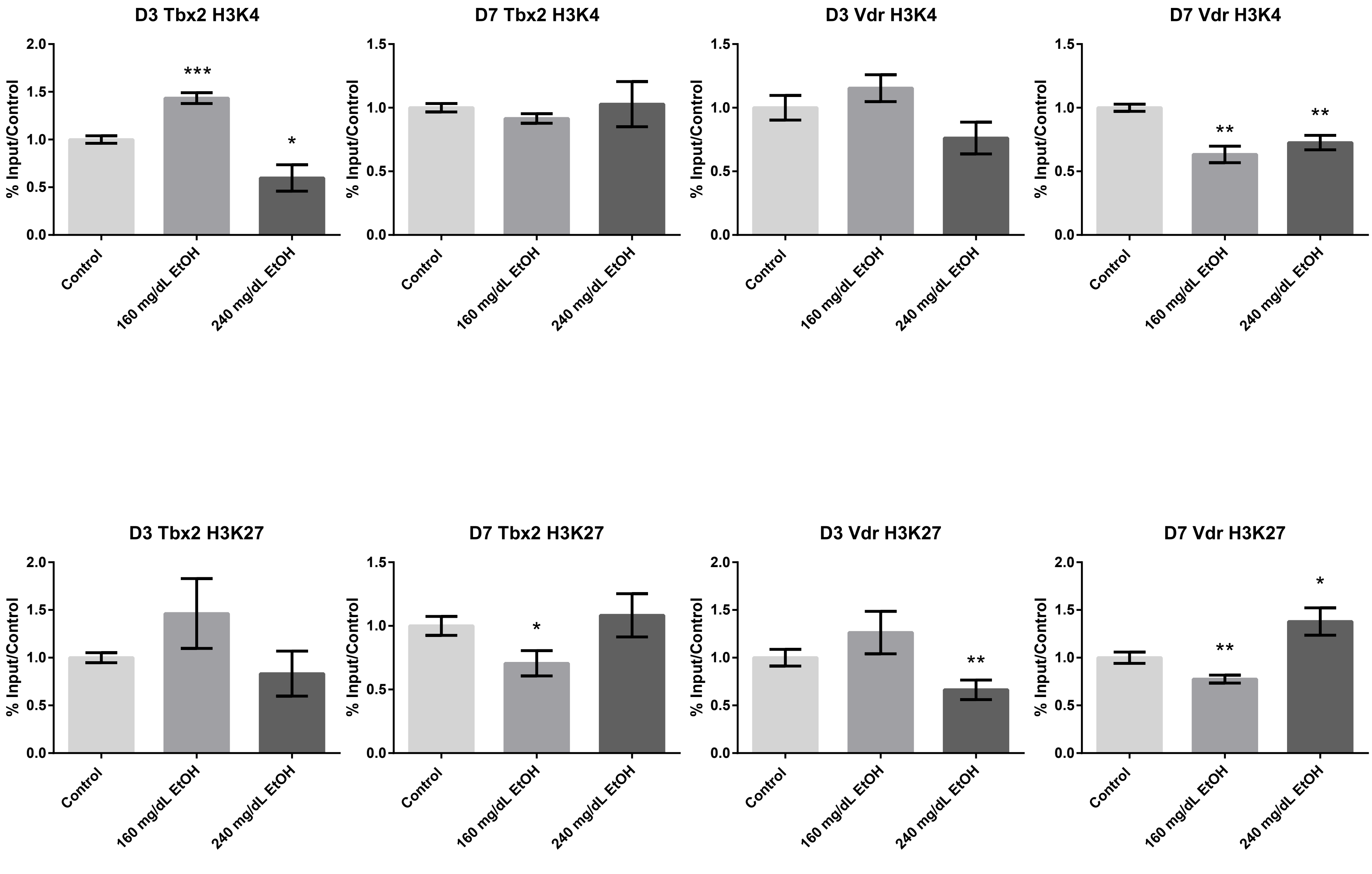
D3 Tbx2 H3K4
D7 Tbx2 H3K4
D3 Vdr H3K4
D7 Vdr H3K4
D3 Tbx2 H3K27
D7 Tbx2 H3K27
D3 Vdr H3K27
D7 Vdr H3K27
% Input/Control
0.0
0.5
1.0
1.5
2.0
Control
160 mg/dL EtOH
240 mg/dL EtOH
***
**
*

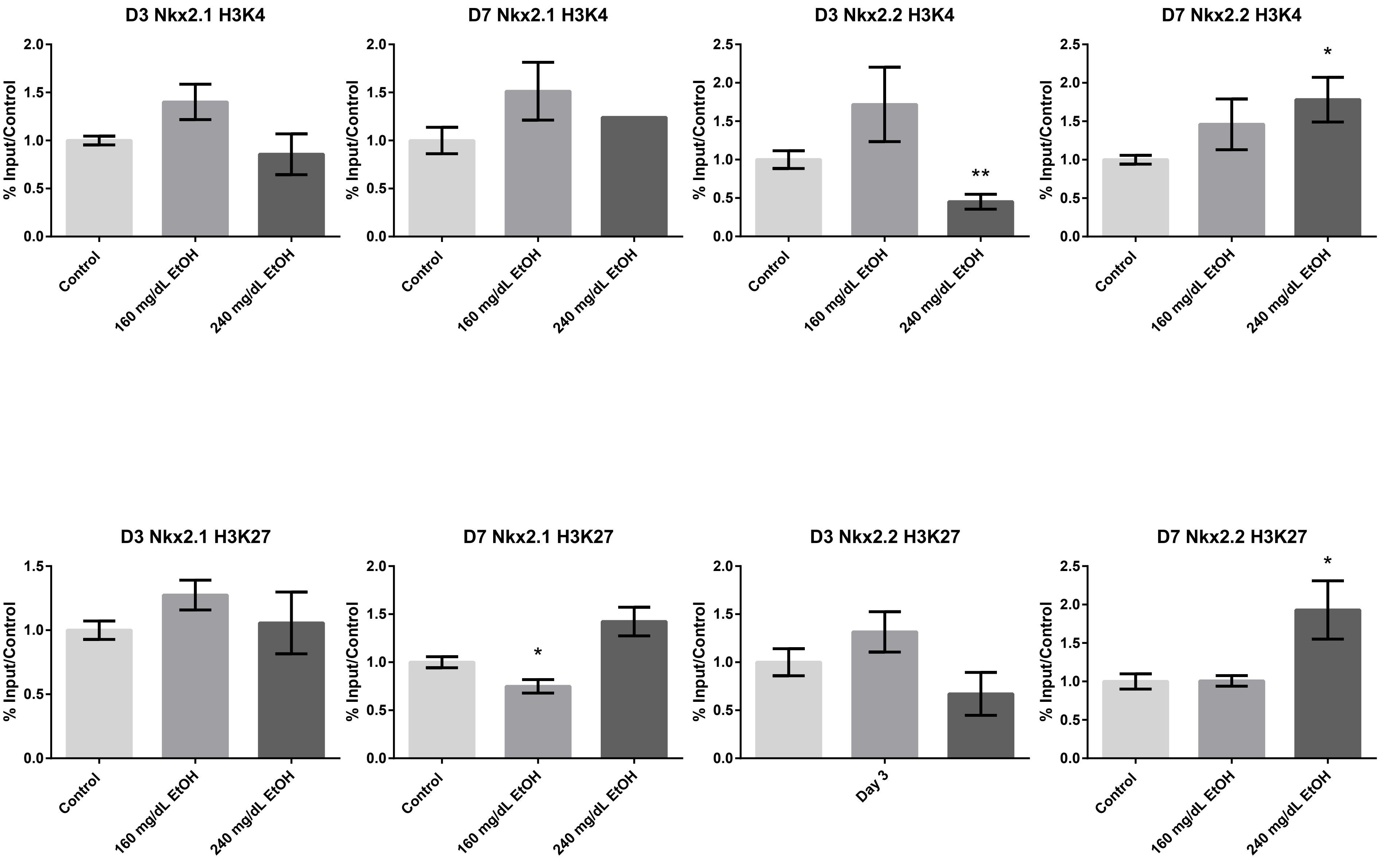
D3 Nkx2.1 H3K4
D7 Nkx2.1 H3K4
D3 Nkx2.2 H3K4
D7 Nkx2.2 H3K4
D3 Nkx2.1 H3K27
D7 Nkx2.1 H3K27
D3 Nkx2.2 H3K27
D7 Nkx2.2 H3K27
% Input/Control
0.0
0.5
1.0
1.5
2.0
2.5
Control
160 mg/dL EtOH
240 mg/dL EtOH
Day 3
*
**

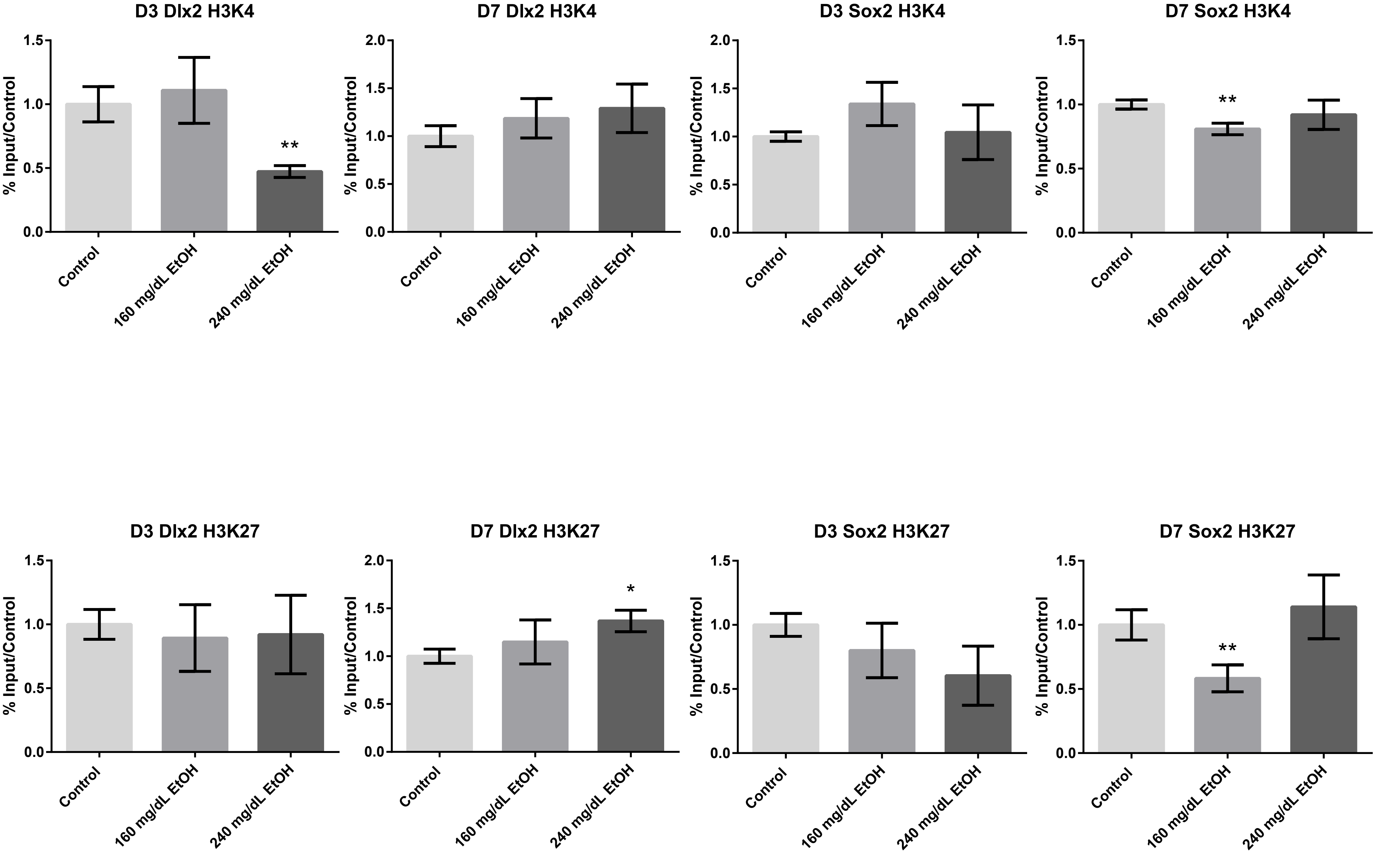

D3 Dlx2 H3K4
D7 Dlx2 H3K4
D3 Sox2 H3K4
D7 Sox2 H3K4
D3 Dlx2 H3K27
D7 Dlx2 H3K27
D3 Sox2 H3K27
D7 Sox2 H3K27
% Input/Control
0.0
0.5
1.0
1.5
2.0
Control
160 mg/dL EtOH
240 mg/dL EtOH
**
*

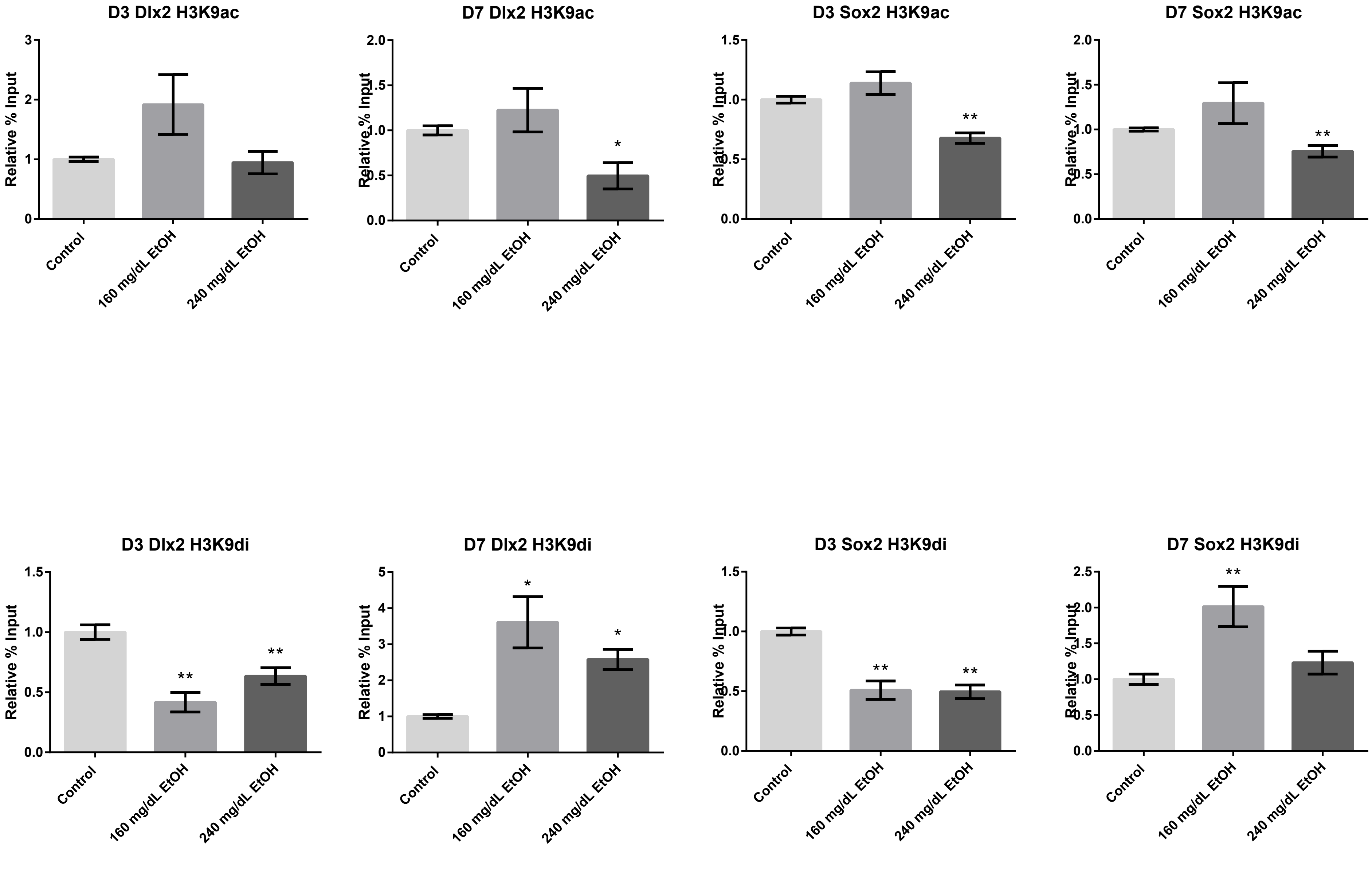
D3 Dlx2 H3K9ac
Relative % Input
0
1
2
3
Control
160 mg/dL EtOH
240 mg/dL EtOH
D7 Dlx2 H3K9ac
Relative % Input
0.0
0.5
1.0
1.5
2.0
*
Control
160 mg/dL EtOH
240 mg/dL EtOH
D3 Sox2 H3K9ac
Relative % Input
0.0
0.5
1.0
1.5
**
Control
160 mg/dL EtOH
240 mg/dL EtOH
D7 Sox2 H3K9ac
Relative % Input
0.0
0.5
1.0
1.5
2.0
**
Control
160 mg/dL EtOH
240 mg/dL EtOH
D3 Dlx2 H3K9di
Relative % Input
0.0
0.5
1.0
1.5
**
**
Control
160 mg/dL EtOH
240 mg/dL EtOH
D7 Dlx2 H3K9di
Relative % Input
0
1
2
3
4
5
*
*
Control
160 mg/dL EtOH
240 mg/dL EtOH
D3 Sox2 H3K9di
Relative % Input
0.0
0.5
1.0
1.5
**
**
Control
160 mg/dL EtOH
240 mg/dL EtOH
D7 Sox2 H3K9di
Relative % Input
0.0
0.5
1.0
1.5
2.0
2.5
**
Control
160 mg/dL EtOH
240 mg/dL EtOH

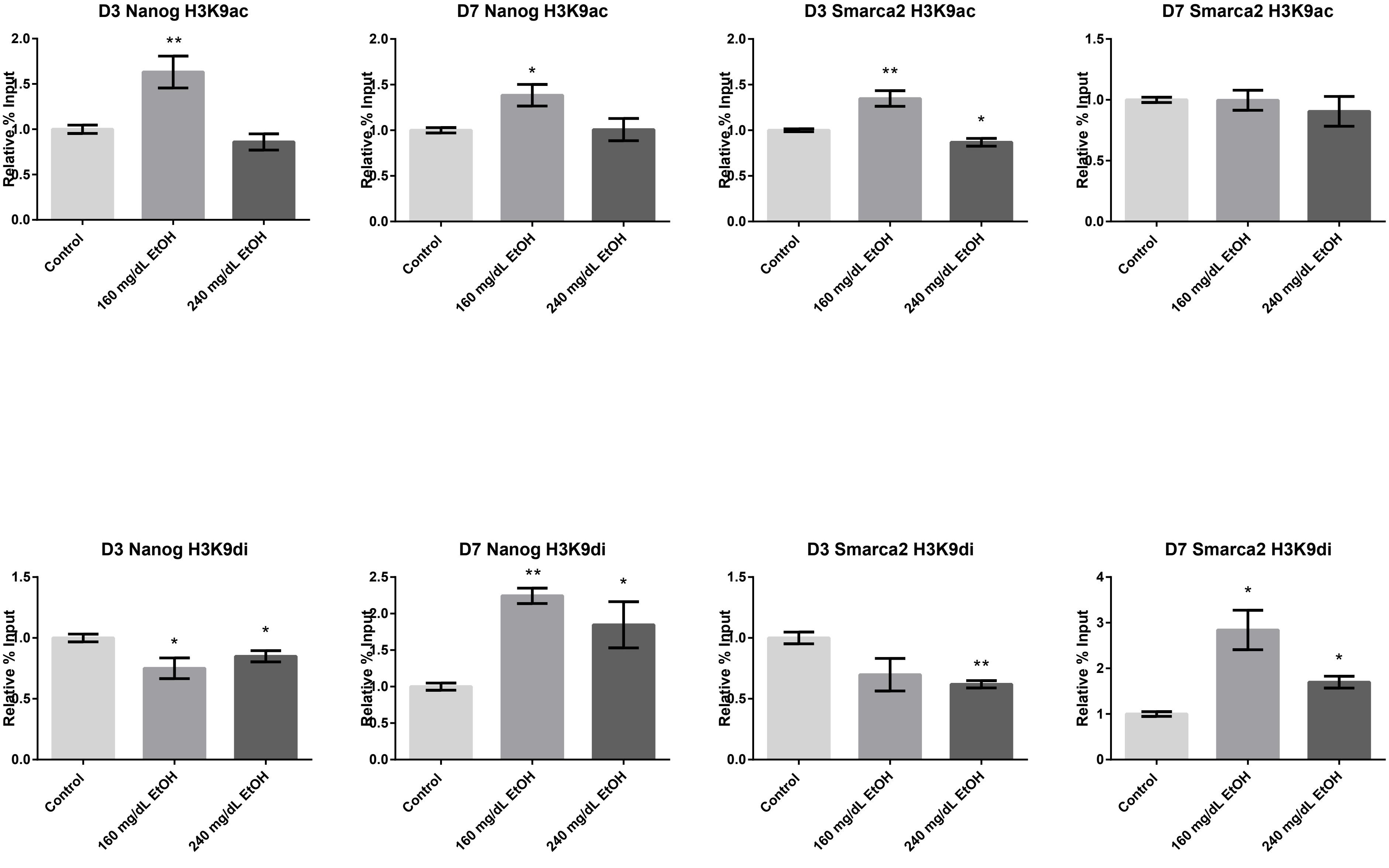
D3 Nanog H3K9ac
D7 Nanog H3K9ac
D3 Smarca2 H3K9ac
D7 Smarca2 H3K9ac
D3 Nanog H3K9di
D7 Nanog H3K9di
D3 Smarca2 H3K9di
D7 Smarca2 H3K9di
Relative % Input
Control
160 mg/dL EtOH
240 mg/dL EtOH
*
**

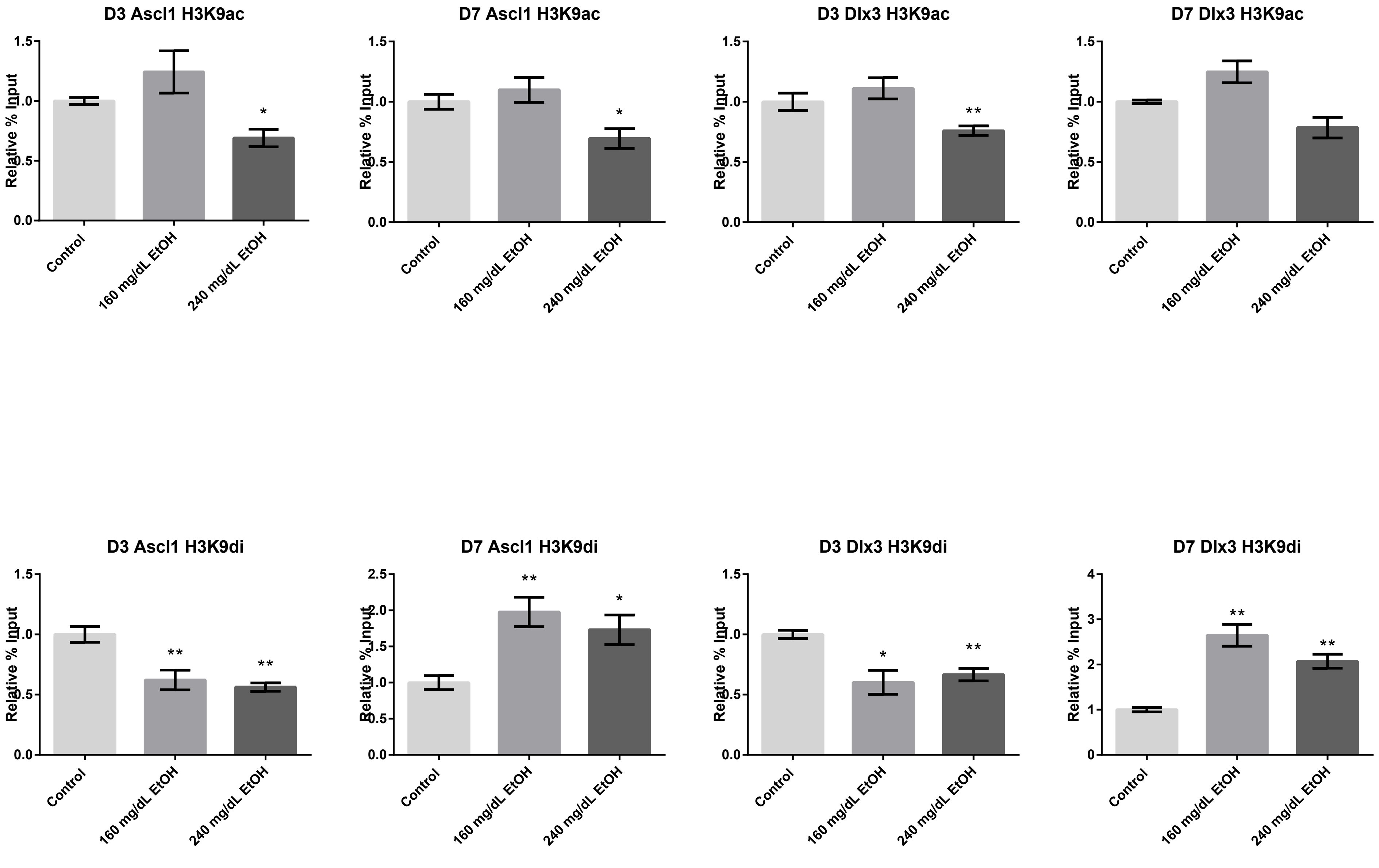

D3 Ascl1 H3K9ac
D7 Ascl1 H3K9ac
D3 Dlx3 H3K9ac
D7 Dlx3 H3K9ac
D3 Ascl1 H3K9di
D7 Ascl1 H3K9di
D3 Dlx3 H3K9di
D7 Dlx3 H3K9di
Relative % Input
Control
160 mg/dL EtOH
240 mg/dL EtOH
0.0
0.5
1.0
1.5
2.0
2.5
0
1
2
3
4
*
**

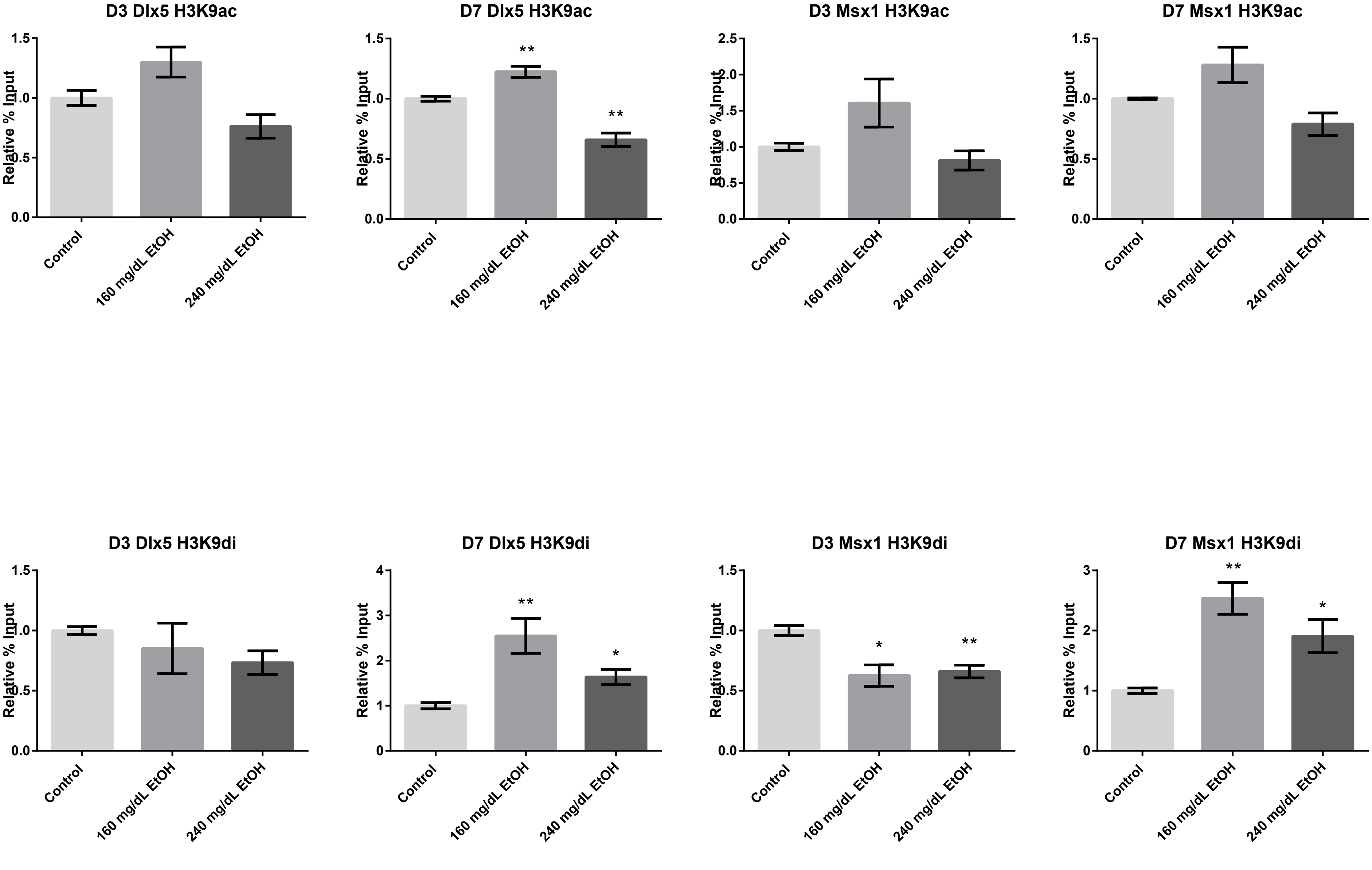
D3 Dlx5 H3K9ac
D7 Dlx5 H3K9ac
D3 Msx1 H3K9ac
D7 Msx1 H3K9ac
D3 Dlx5 H3K9di
D7 Dlx5 H3K9di
D3 Msx1 H3K9di
D7 Msx1 H3K9di
Relative % Input
Control
160 mg/dL EtOH
240 mg/dL EtOH
**
*

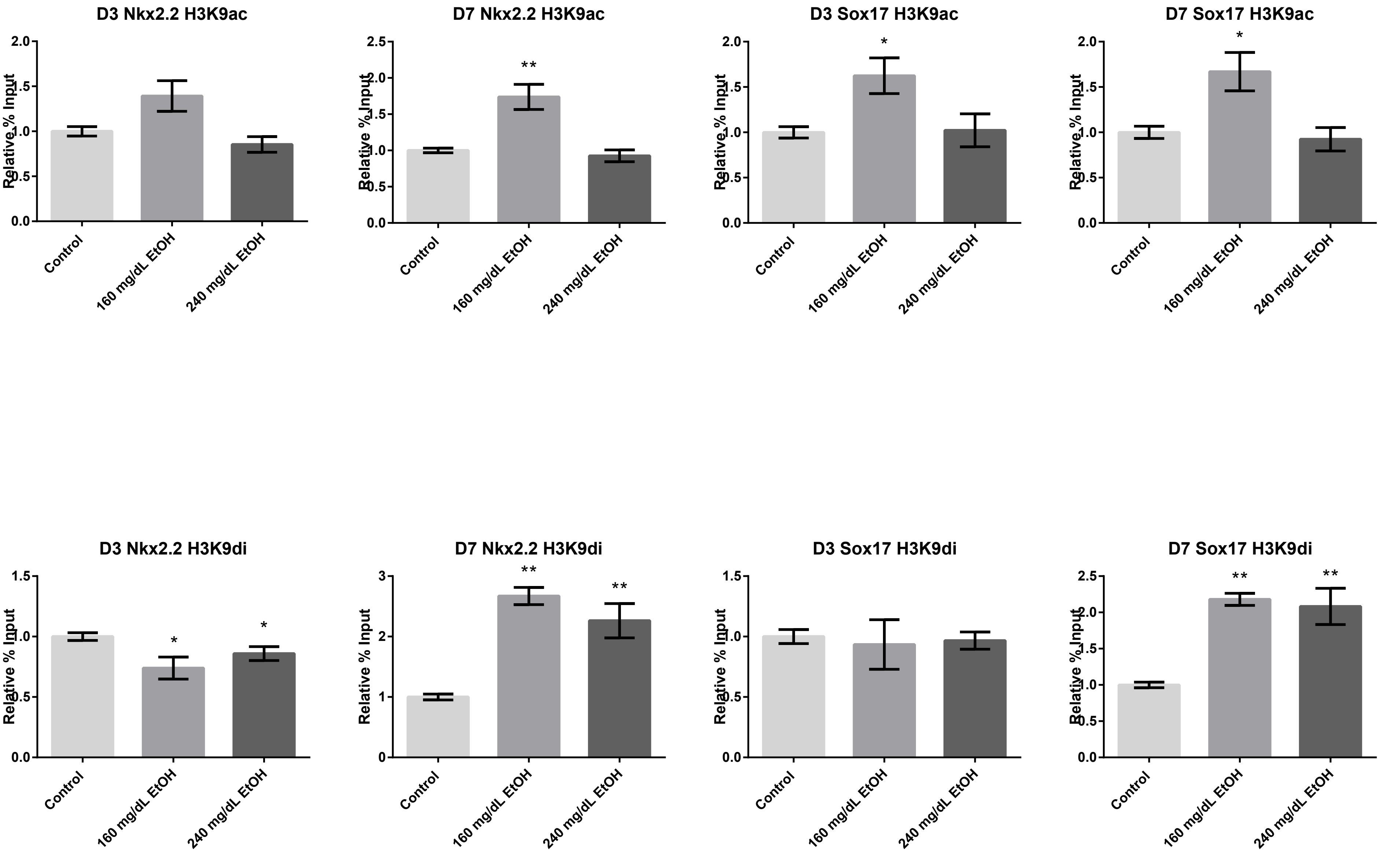
D3 Nkx2.2 H3K9ac
D7 Nkx2.2 H3K9ac
D3 Sox17 H3K9ac
D7 Sox17 H3K9ac
D3 Nkx2.2 H3K9di
D7 Nkx2.2 H3K9di
D3 Sox17 H3K9di
D7 Sox17 H3K9di
Relative % Input
Control
160 mg/dL EtOH
240 mg/dL EtOH
*
**

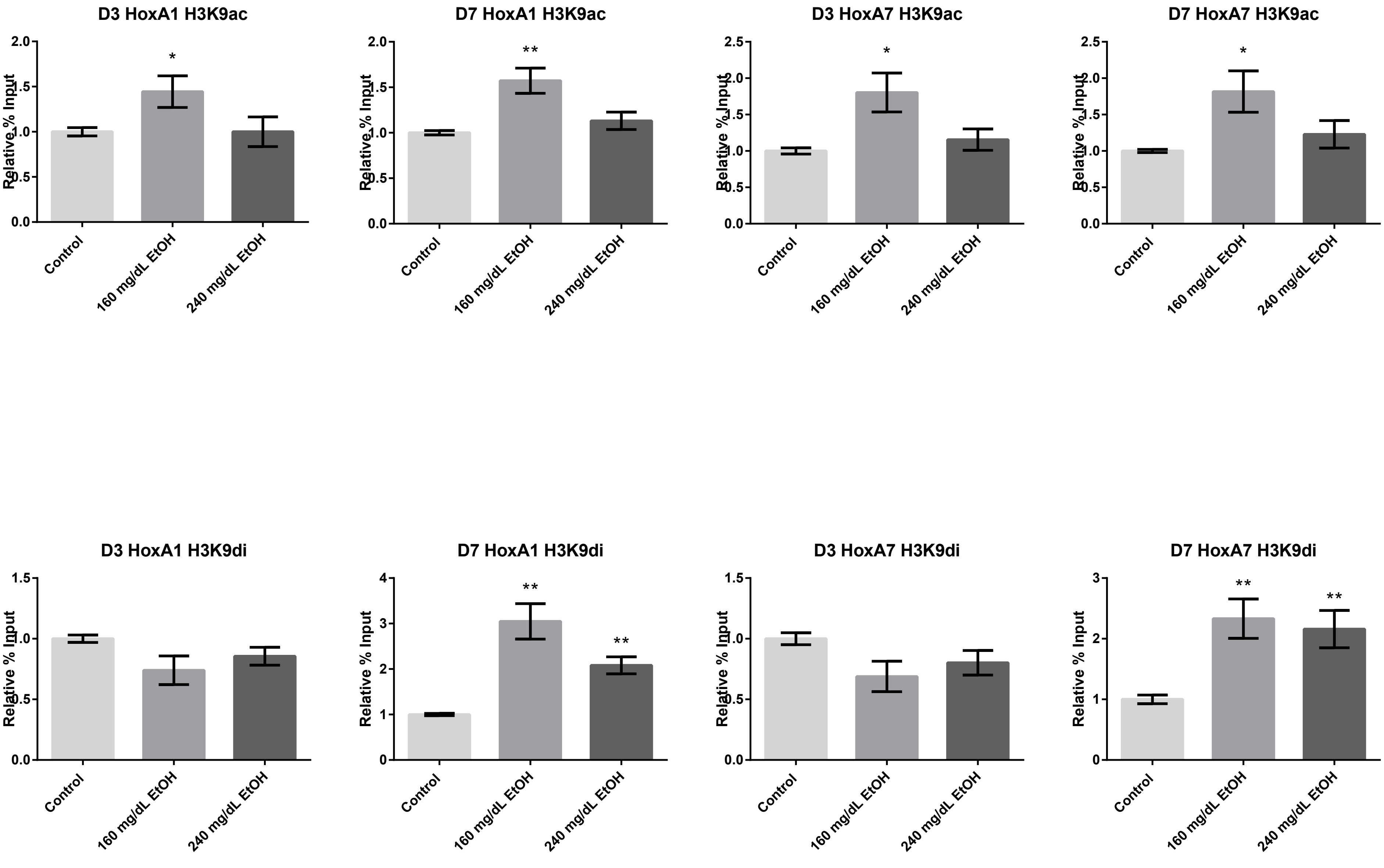
D3 HoxA1 H3K9ac
D7 HoxA1 H3K9ac
D3 HoxA7 H3K9ac
D7 HoxA7 H3K9ac
D3 HoxA1 H3K9di
D7 HoxA1 H3K9di
D3 HoxA7 H3K9di
D7 HoxA7 H3K9di
Relative % Input
Control
160 mg/dL EtOH
240 mg/dL EtOH
*
**

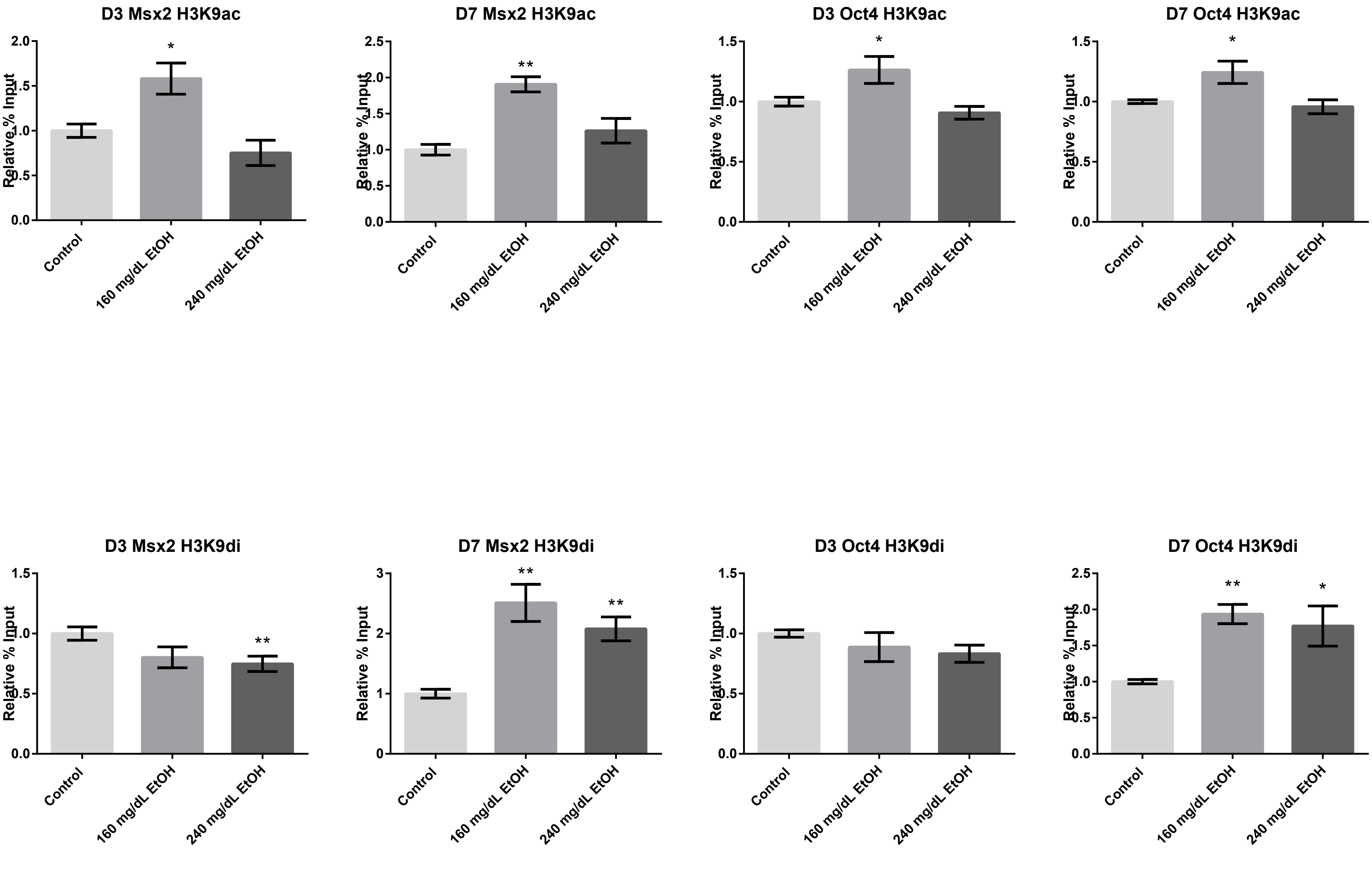

D3 Msx2 H3K9ac
D7 Msx2 H3K9ac
D3 Oct4 H3K9ac
D7 Oct4 H3K9ac
D3 Msx2 H3K9di
D7 Msx2 H3K9di
D3 Oct4 H3K9di
D7 Oct4 H3K9di
Relative % Input
0.0
0.5
1.0
1.5
2.0
2.5
0
1
2
3
Control
160 mg/dL EtOH
240 mg/dL EtOH
*
**

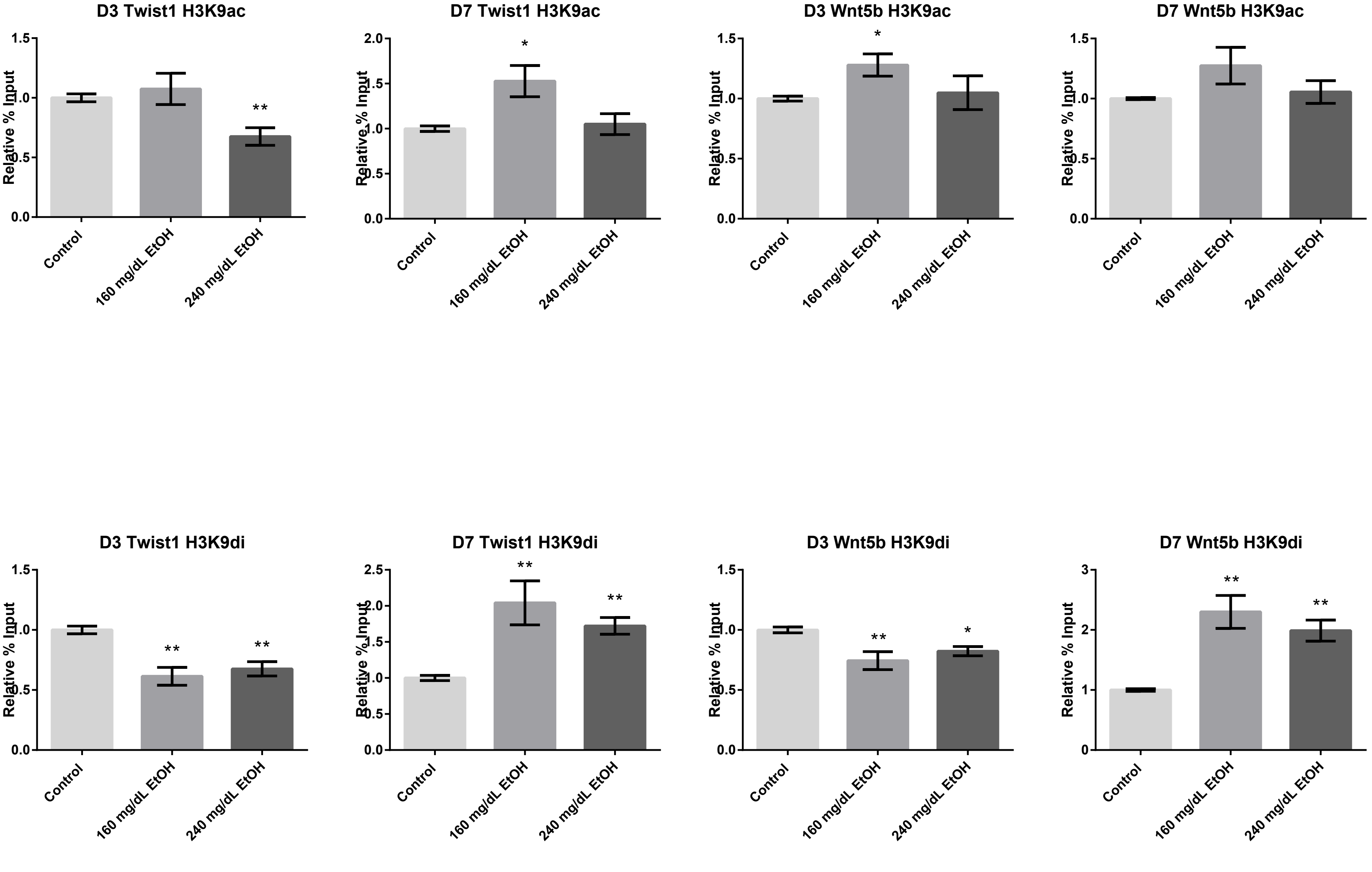

D3 Twist1 H3K9ac
D7 Twist1 H3K9ac
D3 Wnt5b H3K9ac
D7 Wnt5b H3K9ac
D3 Twist1 H3K9di
D7 Twist1 H3K9di
D3 Wnt5b H3K9di
D7 Wnt5b H3K9di
Relative % Input
Control
160 mg/dL EtOH
240 mg/dL EtOH

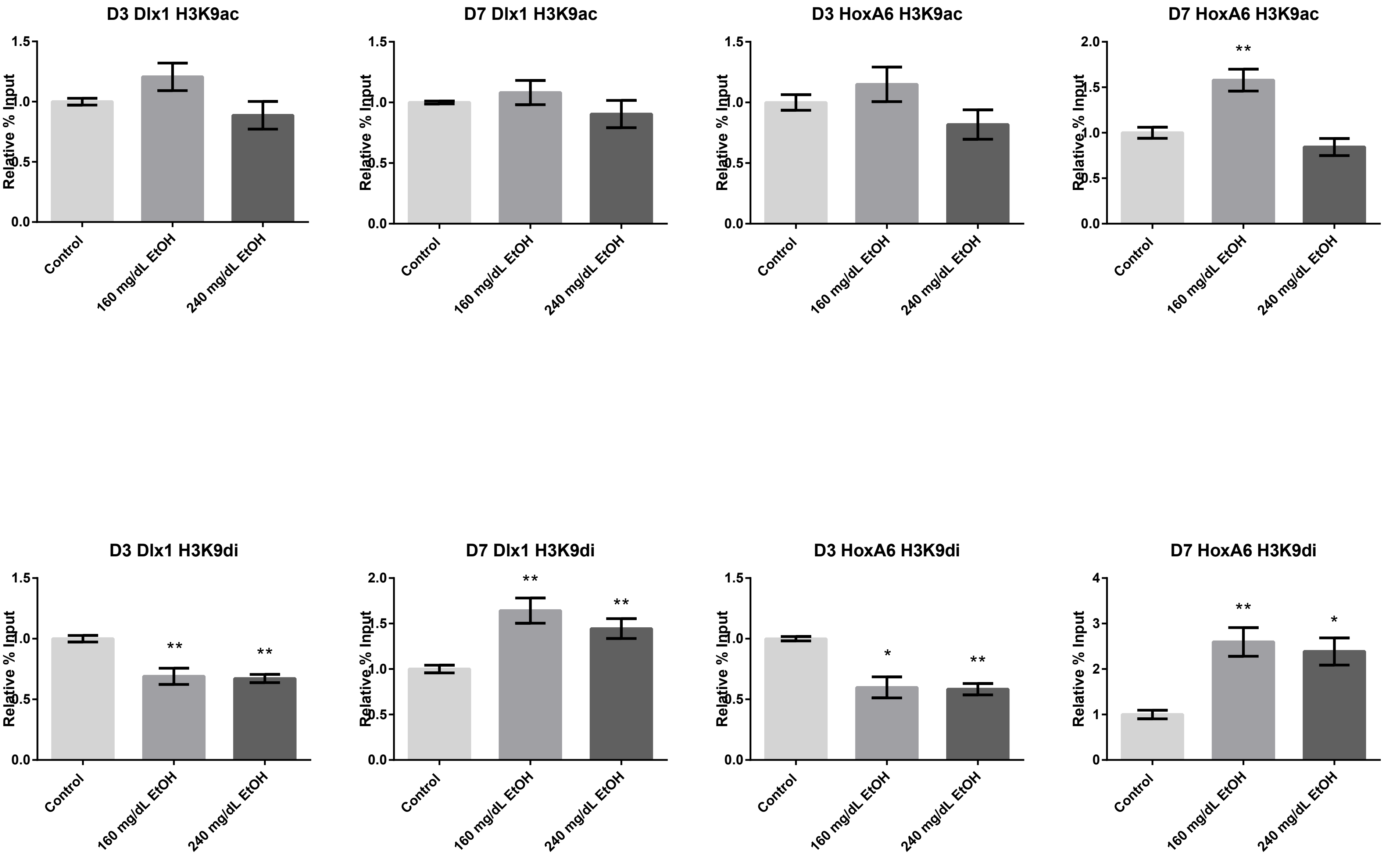
D3 Dlx1 H3K9ac
D7 Dlx1 H3K9ac
D3 HoxA6 H3K9ac
D7 HoxA6 H3K9ac
D3 Dlx1 H3K9di
D7 Dlx1 H3K9di
D3 HoxA6 H3K9di
D7 HoxA6 H3K9di
Relative % Input
0.0
0.5
1.0
1.5
2.0
0
1
2
3
4
Control
160 mg/dL EtOH
240 mg/dL EtOH
**
*

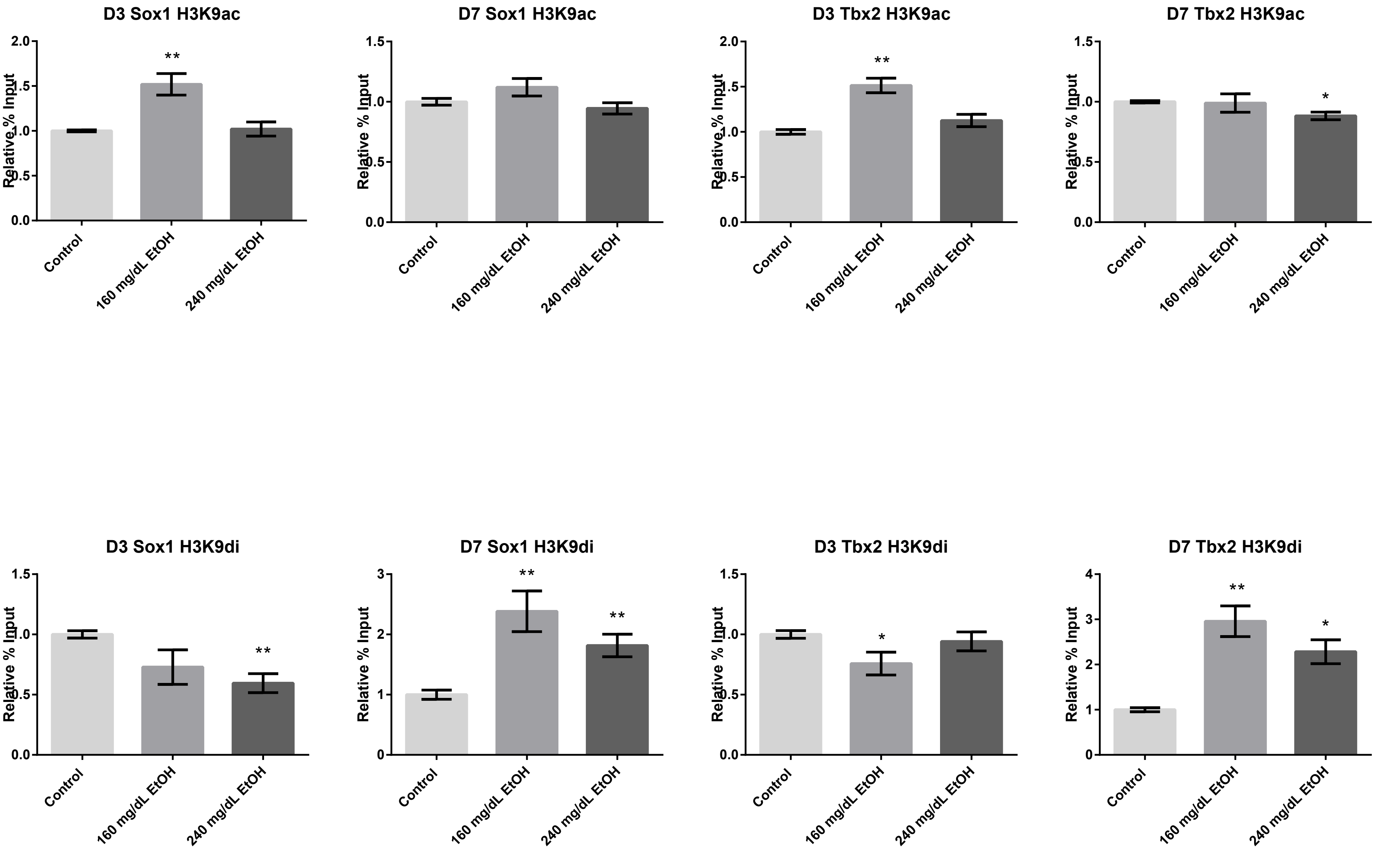
D3 Sox1 H3K9ac
D7 Sox1 H3K9ac
D3 Tbx2 H3K9ac
D7 Tbx2 H3K9ac
D3 Sox1 H3K9di
D7 Sox1 H3K9di
D3 Tbx2 H3K9di
D7 Tbx2 H3K9di
Relative % Input
Control
160 mg/dL EtOH
240 mg/dL EtOH
**
*

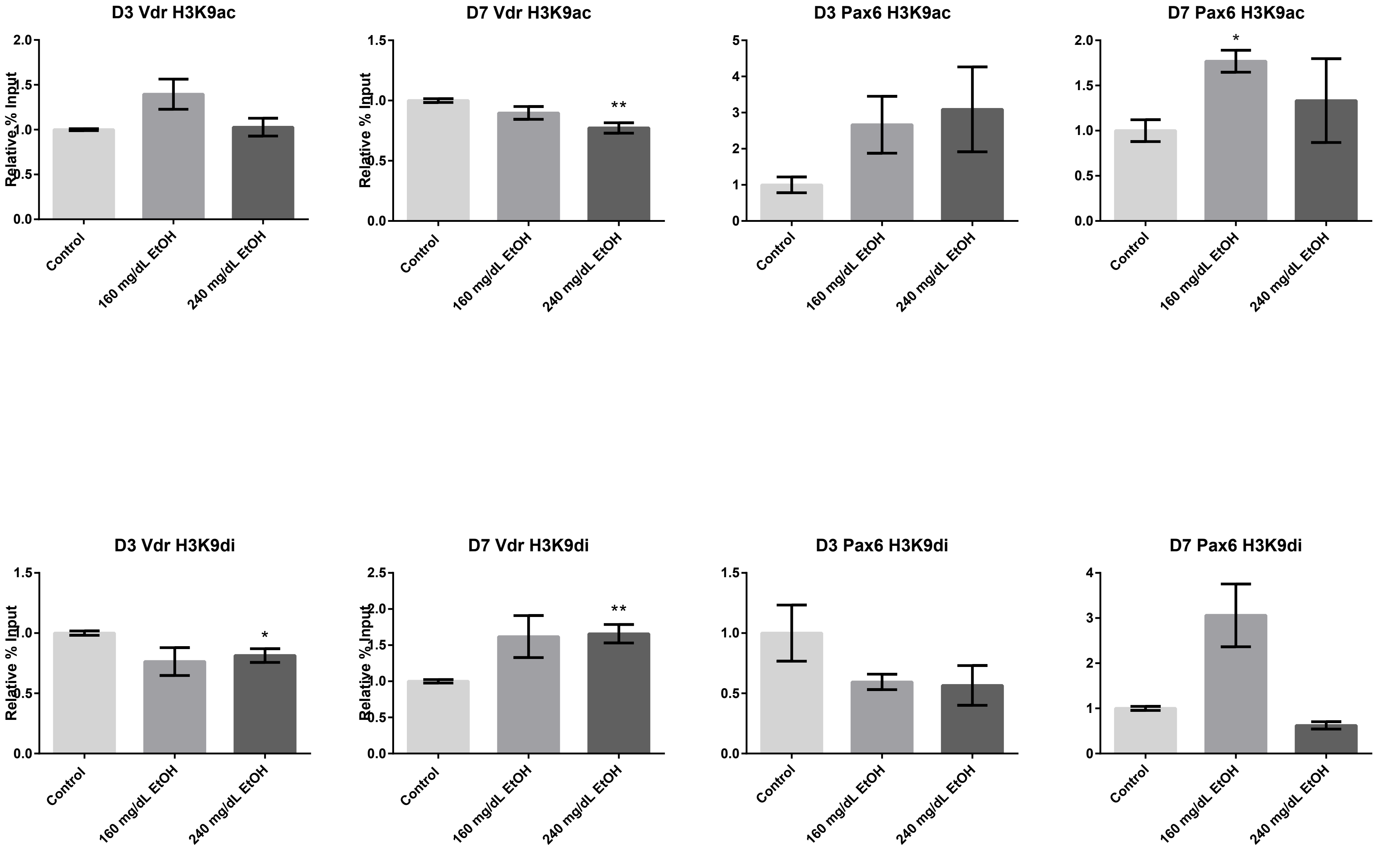

D3 Vdr H3K9ac
Relative % Input
2.0
1.5
1.0
0.5
0.0
Control
160 mg/dL EtOH
240 mg/dL EtOH
D7 Vdr H3K9ac
Relative % Input
1.5
1.0
0.5
0.0
**
Control
160 mg/dL EtOH
240 mg/dL EtOH
D3 Pax6 H3K9ac
5
4
3
2
1
0
Control
160 mg/dL EtOH
240 mg/dL EtOH
D7 Pax6 H3K9ac
2.0
1.5
1.0
0.5
0.0
*
Control
160 mg/dL EtOH
240 mg/dL EtOH
D3 Vdr H3K9di
Relative % Input
1.5
1.0
0.5
0.0
*
Control
160 mg/dL EtOH
240 mg/dL EtOH
D7 Vdr H3K9di
Relative % Input
2.5
2.0
1.5
1.0
0.5
0.0
**
Control
160 mg/dL EtOH
240 mg/dL EtOH
D3 Pax6 H3K9di
1.5
1.0
0.5
0.0
Control
160 mg/dL EtOH
240 mg/dL EtOH
D7 Pax6 H3K9di
4
3
2
1
0
Control
160 mg/dL EtOH
240 mg/dL EtOH
