# Supplementary material for: Dose-dependent alcohol-induced alterations in chromatin structure persist beyond the window of exposure and correlate with fetal alcohol syndrome birth defects
Source: Epigenetics Chromatin. 2015 Sep 28;8:39. doi: 10.1186/s13072-015-0031-7 (PMC4587584; doi:10.1186/s13072-015-0031-7)
Supplement: Supplementary file 2 — 10.1186/s13072-015-0031-7 ChIP assay and quantitative PCR (qPCR) analysis of the individual candidate genes examined in Figure 7. [file 13072_2015_31_MOESM2_ESM.pdf]

Additional Figure 2

Lasting alcohol-induced alterations in H3K4 me3, H3K9 ac, H3K9 me2, and H3K27 me3 within the prenatal cortex arising from an early gestational exposure.

Pregnant dams were injected with 2.9g/kg EtOH at GD7 and embryos harvested at GD17. Embryos were scored for ocular and cortical patterning defects, and sorted into 3 groups - control, EtOH exposed - morphologically normal, and EtOH exposed - malformed. After dissection of the fetal cortex, ChIP-qPCR analysis was performed on cellular extracts using antibodies recognizing H3K4 me3, H3K9 ac, H3K9 me2, and H3K27me3. In the experiments examining H3K4 me3 and H3K27 me3, four ChIP experiments were performed on a total of 15 brains across 5 different litters. For analysis of H3K9 ac and H3K9 me2, three ChIP experiments were performed on a total of 10 brains across 5 different litters. Primers were designed to fall within 250 base pairs of the transcriptional start site. Results are graphed relative to 1% of the total input and for each sample, background levels are represented by the IgG control. Two replicates of qPCR were performed on each ChIP. Significance was determined using a two-way ANOVA. Error bars represent SEM.*$p<0.05$; **$p<0.01$; ***$p<0.001$; ****$p<0.0001$.

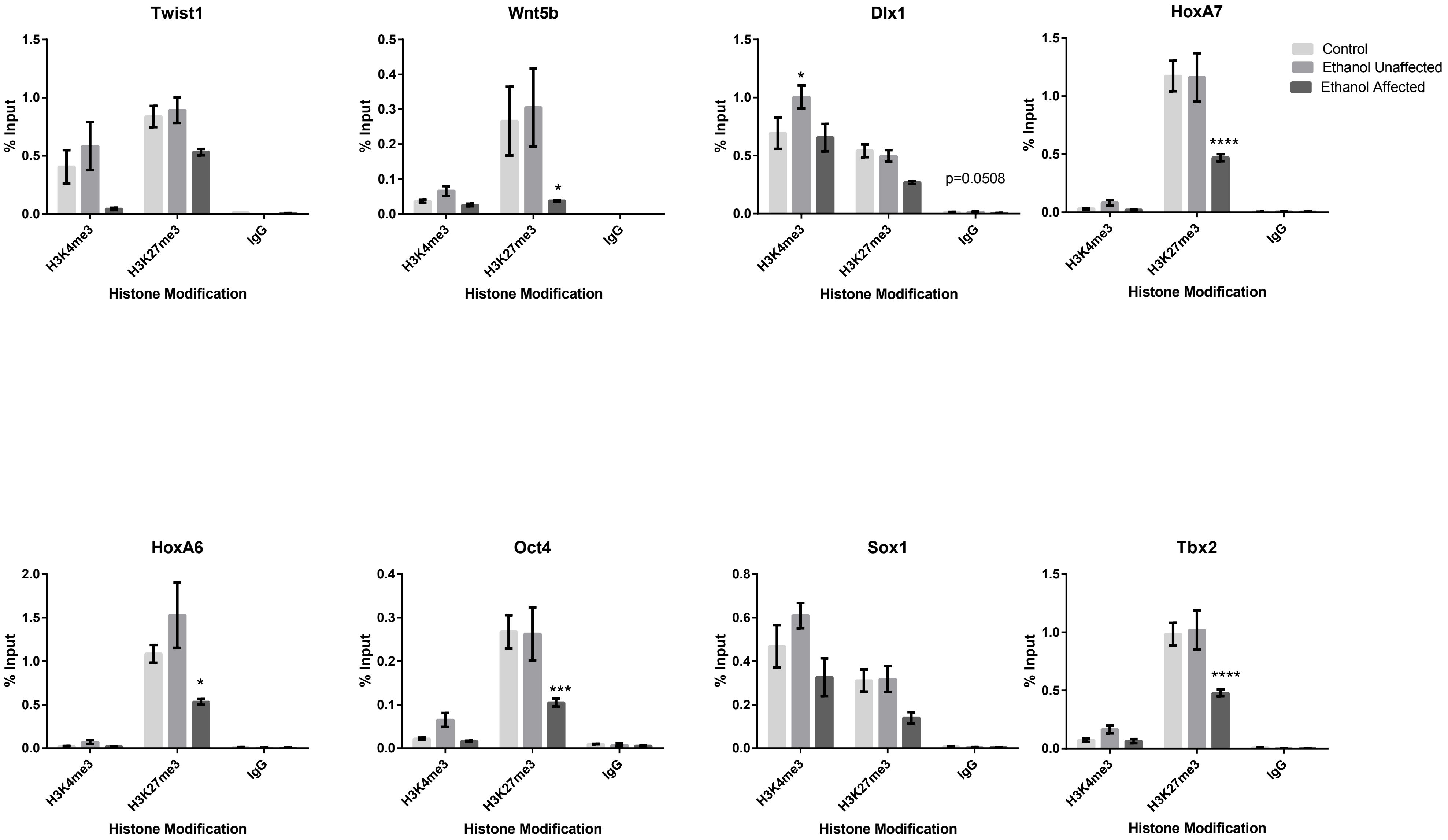
Twist1
Wnt5b
Dlx1
HoxA7
HoxA6
Oct4
Sox1
Tbx2
Control
Ethanol Unaffected
Ethanol Affected
% Input
Histone Modification
H3K4me3
H3K27me3
IgG
p=0.0508
*
***
****

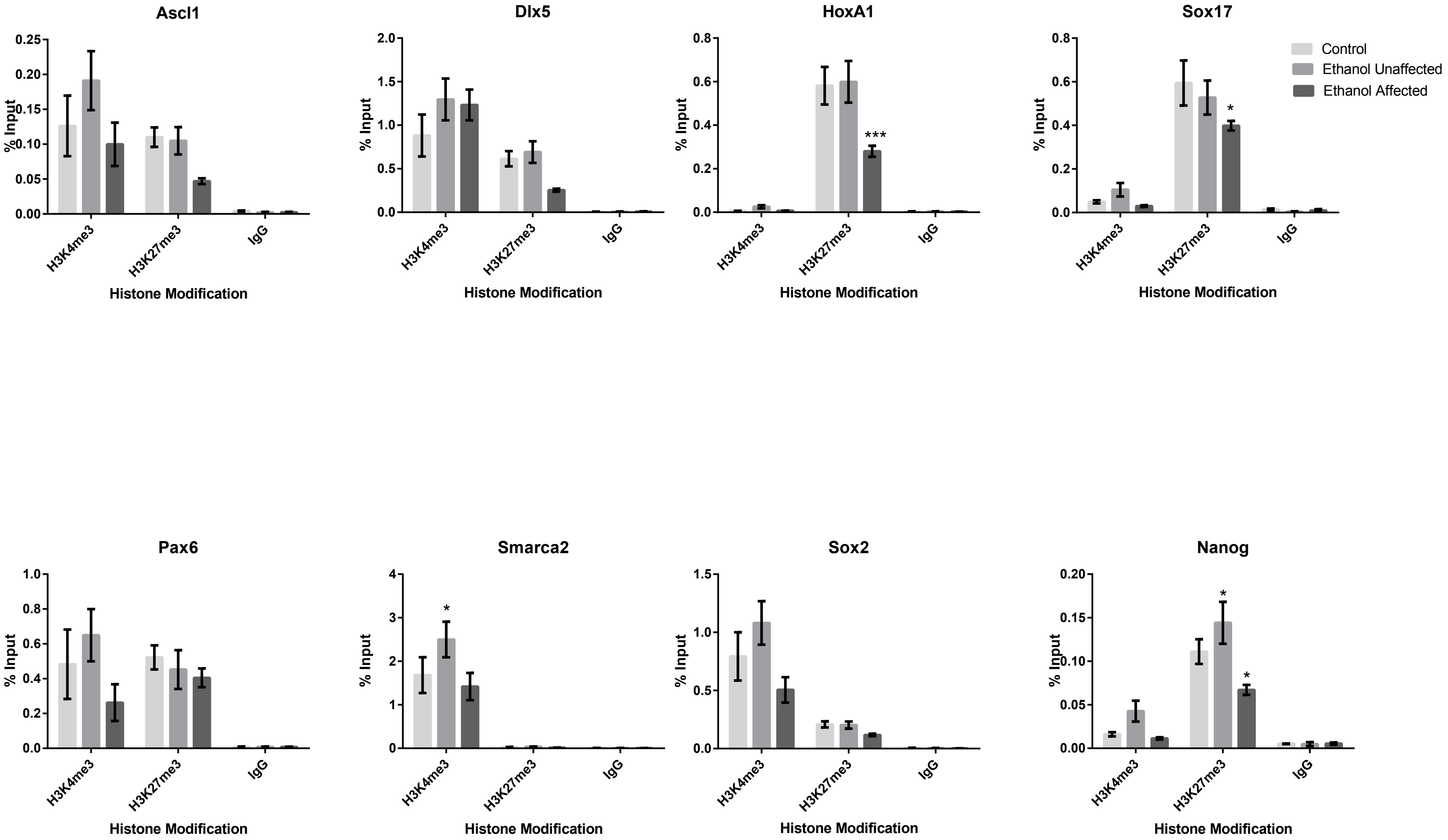
Ascl1
Dlx5
HoxA1
Sox17
Control
Ethanol Unaffected
Ethanol Affected
% Input
Histone Modification
H3K4me3
H3K27me3
IgG
***
*
Pax6
Smarca2
Sox2
Nanog

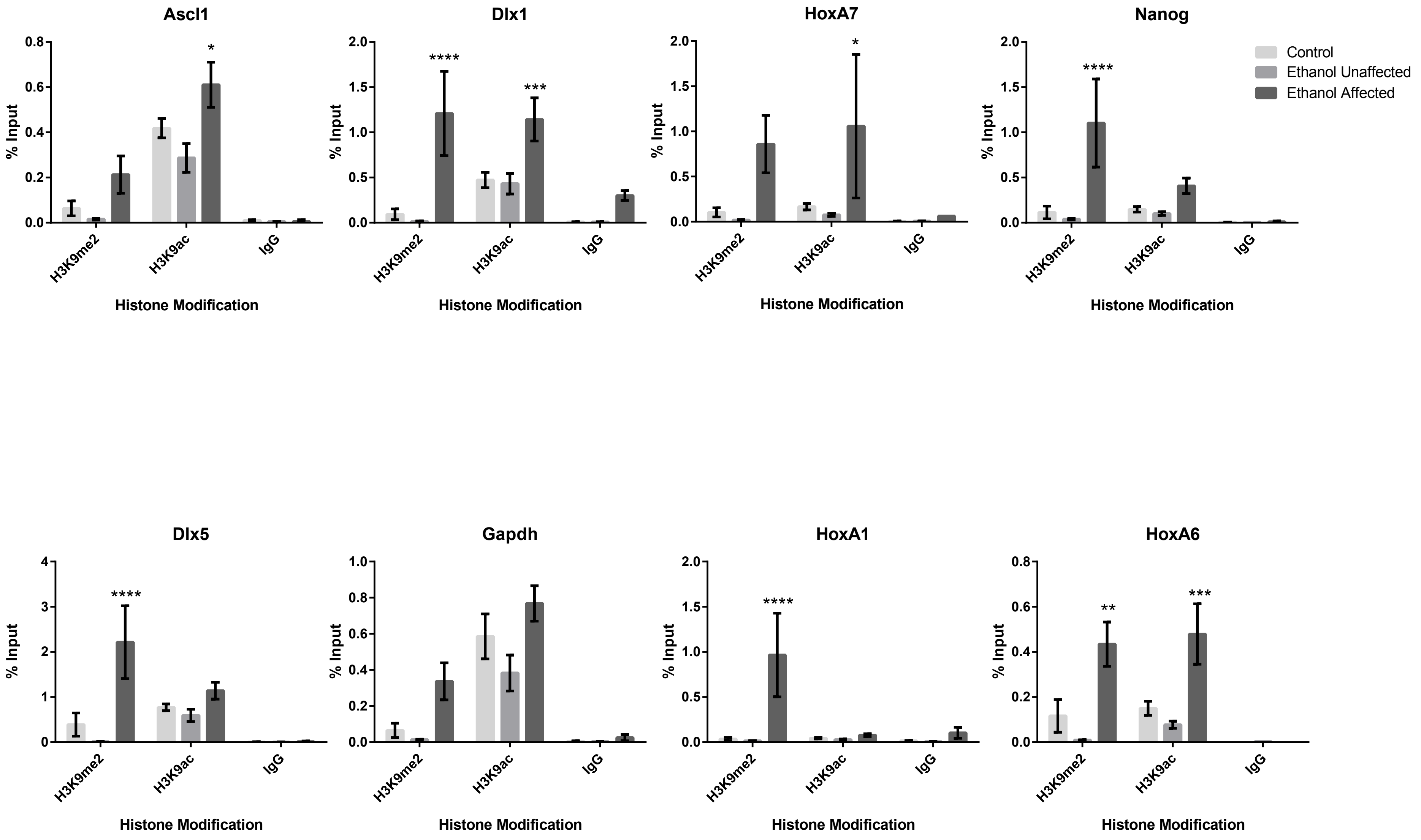
Ascl1
Dlx1
HoxA7
Nanog
Dlx5
Gapdh
HoxA1
HoxA6
% Input
Histone Modification
H3K9me2
H3K9ac
IgG
Control
Ethanol Unaffected
Ethanol Affected

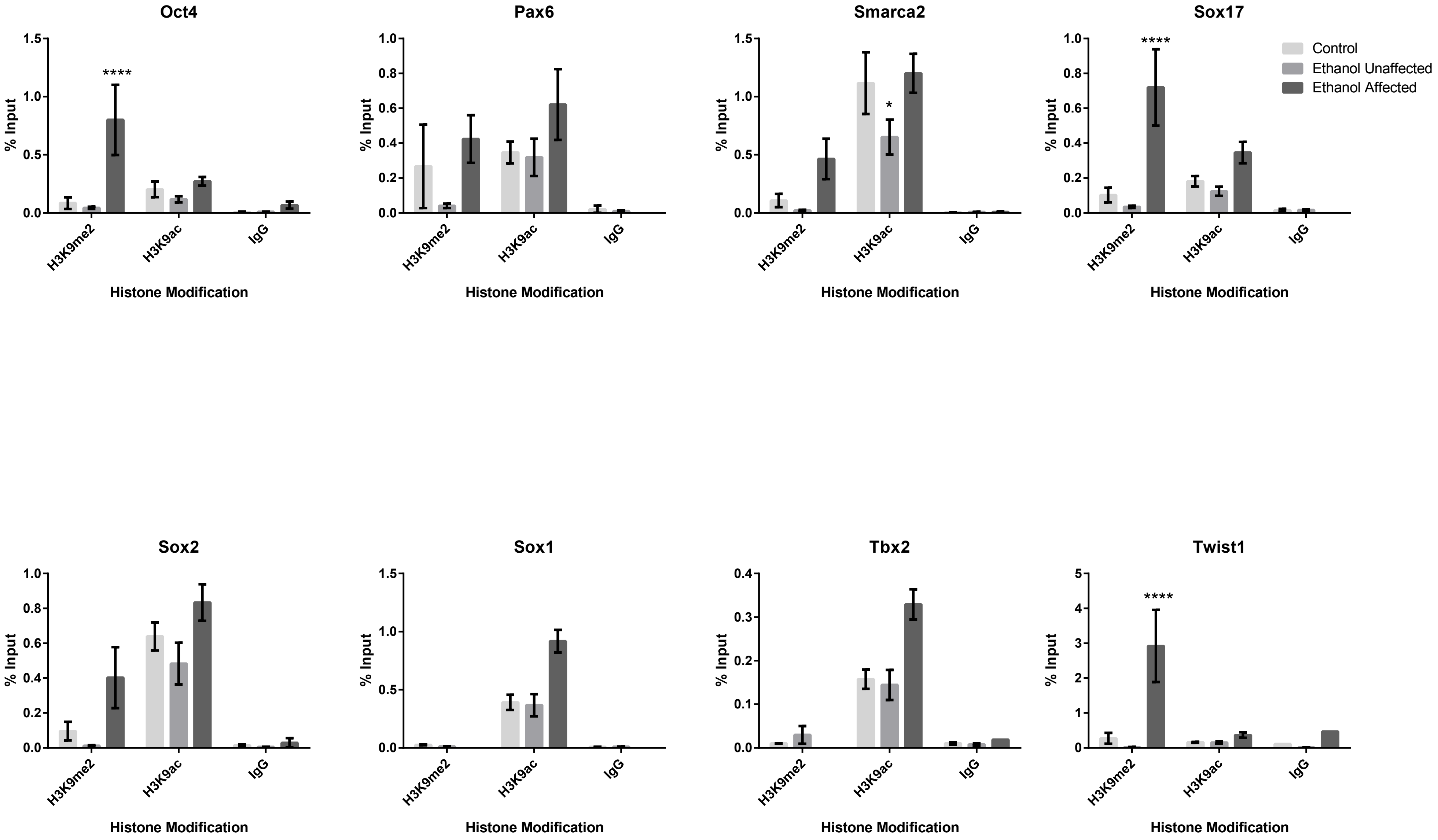
Oct4
Pax6
Smarca2
Sox17
Sox2
Sox1
Tbx2
Twist1
Control
Ethanol Unaffected
Ethanol Affected
% Input
H3K9me2
H3K9ac
IgG
Histone Modification
****
*
